# Strategies to save energy in the context of the energy crisis: a review

**DOI:** 10.1007/s10311-023-01591-5

**Published:** 2023-03-23

**Authors:** Mohamed Farghali, Ahmed I. Osman, Israa M. A. Mohamed, Zhonghao Chen, Lin Chen, Ikko Ihara, Pow-Seng Yap, David W. Rooney

**Affiliations:** 1 Department of Agricultural Engineering and Socio-Economics, Kobe University, Kobe, 657-8501 Japan; 2 Department of Animal and Poultry Hygiene and Environmental Sanitation, Faculty of Veterinary Medicine, Assiut University, Assiut, 71526 Egypt; 3 School of Chemistry and Chemical Engineering, Queen’s University Belfast, David Keir Building, Stranmillis Road, Belfast, BT9 5AG Northern Ireland UK; 4 Department of Civil Engineering, Xi’an Jiaotong-Liverpool University, Suzhou, 215123 China; 5 School of Civil Engineering, Chongqing University, Chongqing, 400045 China; 6 Key Laboratory of New Technology for Construction of Cities in Mountain Area, Ministry of Education, Chongqing University, Chongqing, 400045 China

**Keywords:** Energy saving, Energy crisis, Green energy alternative, Artificial intelligence, Electric vehicles, Renewable energy

## Abstract

New technologies, systems, societal organization and policies for energy saving are urgently needed in the context of accelerated climate change, the Ukraine conflict and the past coronavirus disease 2019 pandemic. For instance, concerns about market and policy responses that could lead to new lock-ins, such as investing in liquefied natural gas infrastructure and using all available fossil fuels to compensate for Russian gas supply cuts, may hinder decarbonization efforts. Here we review energy-saving solutions with a focus on the actual energy crisis, green alternatives to fossil fuel heating, energy saving in buildings and transportation, artificial intelligence for sustainable energy, and implications for the environment and society. Green alternatives include biomass boilers and stoves, hybrid heat pumps, geothermal heating, solar thermal systems, solar photovoltaics systems into electric boilers, compressed natural gas and hydrogen. We also detail case studies in Germany which is planning a 100% renewable energy switch by 2050 and developing the storage of compressed air in China, with emphasis on technical and economic aspects. The global energy consumption in 2020 was 30.01% for the industry, 26.18% for transport, and 22.08% for residential sectors. 10–40% of energy consumption can be reduced using renewable energy sources, passive design strategies, smart grid analytics, energy-efficient building systems, and intelligent energy monitoring. Electric vehicles offer the highest cost-per-kilometer reduction of 75% and the lowest energy loss of 33%, yet battery-related issues, cost, and weight are challenging. 5–30% of energy can be saved using automated and networked vehicles. Artificial intelligence shows a huge potential in energy saving by improving weather forecasting and machine maintenance and enabling connectivity across homes, workplaces, and transportation. For instance, 18.97–42.60% of energy consumption can be reduced in buildings through deep neural networking. In the electricity sector, artificial intelligence can automate power generation, distribution, and transmission operations, balance the grid without human intervention, enable lightning-speed trading and arbitrage decisions at scale, and eliminate the need for manual adjustments by end-users.

## Introduction

Energy is required for several practical functions, including transportation, mobility, food preparation, water purification, communication, and others (Kalt et al. 2019; Vahidi and Sciarretta 2018). Global population and economic expansion have contributed to increased energy use over time. The efficiency of energy end-use services has gradually improved due to technological advancement and energy efficiency legislation. Nevertheless, this improvement has not always been adequate to offset the increase in demand for energy services, such as the production and consumption of commodities. In addition, the ongoing conflict between Russia and Ukraine has caused an energy crisis that has directly affected the heating, cooling, and transportation energy costs of households. In 2021, the European Union imported over 45% of its gas and nearly 40% of its total gas consumption (IEA 2022a).

Indirectly, this crisis has increased the prices of additional goods and services across global supply chains. In addition to the current energy crisis, the predominant source of carbon dioxide emissions, the primary greenhouse gas responsible for global warming, is the widespread use of fossil fuels to produce energy (Farghali et al. 2022). Therefore, scholars and decision-makers generally agree that carbon neutrality by the middle of the century and other climate targets outlined in the Paris Agreement can only be achieved by gradually reducing global energy demand to a sustainable level as well as using negative emissions technologies.

**Fig. 1:**
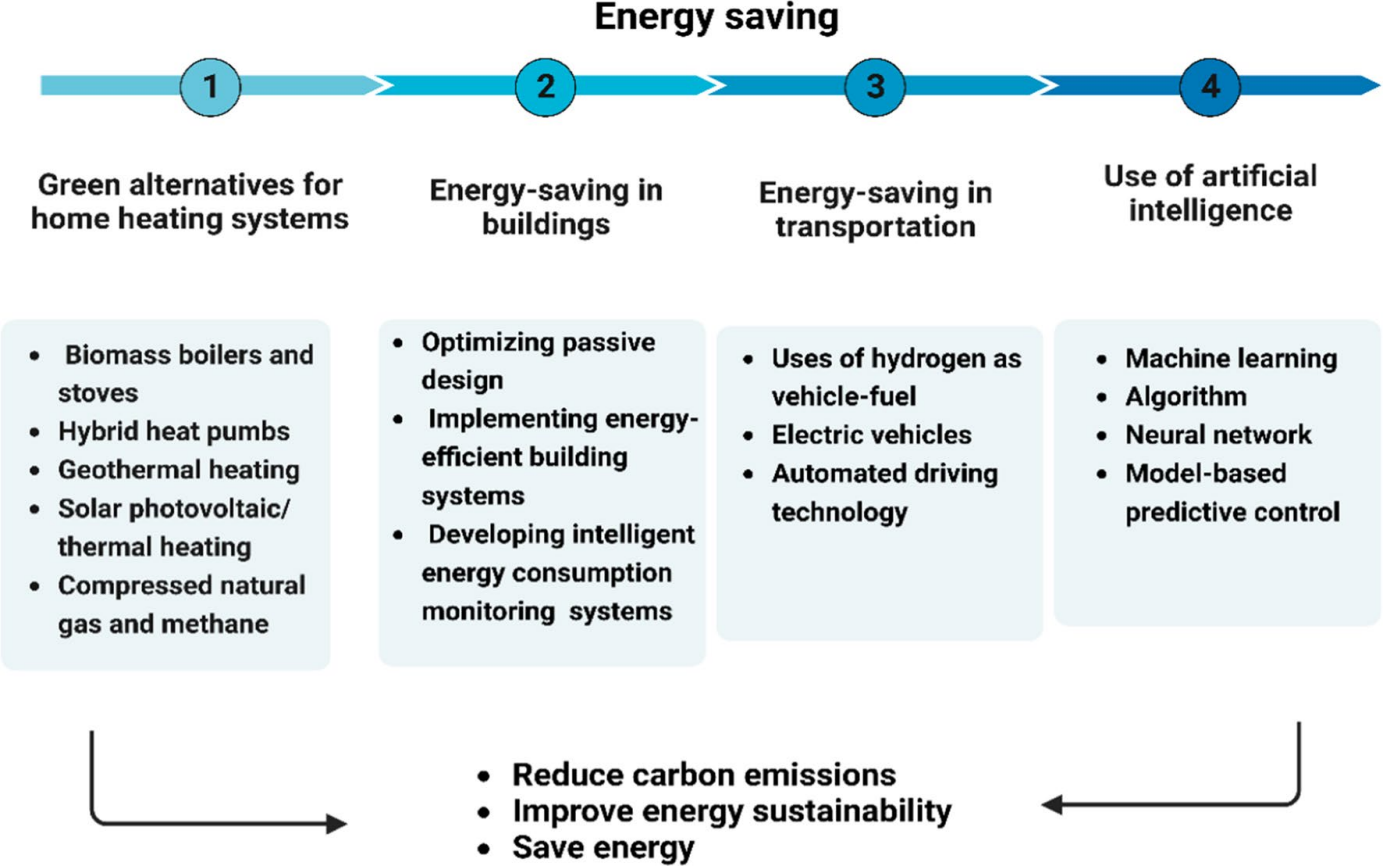
Strategy for energy saving. The review discusses current and new policies that address energy sufficiency and conservation, including progressive home appliances, buildings, and vehicle standards. First, the energy crisis and the potential of renewable energy as providing energy sources are examined. Second, we discuss how to save energy from buildings and vehicles, as well as the uses of electric and hydrogen-powered vehicles in energy saving. Lastly, we identify the role of artificial intelligence in maximizing energy efficiency and achieving significant savings across multiple sectors

Energy savings from energy efficiency and conservation offer additional co-benefits and contribute to national energy supply security and climate change mitigation. These include reducing local pollution, enhancing business competitiveness, lowering household energy costs, boosting productivity, enhancing occupant health in buildings, and helping to lower energy poverty. A change in the behavior and way of life of energy end-users is required to achieve additional energy savings by reducing the need for services, which goes beyond investments in energy efficiency technologies, given the stringency and urgency of the climate target and the current global increase in energy demand. Energy performance criteria for vehicles, appliances, buildings, and financial incentives for new technology, and others have historically been the primary targets and promoters of energy efficiency innovations, as shown in Fig. 1.

In 2016, the combined building and transportation industries of the European Union consumed over 652.1 million tons of oil equivalent, accounting for more than 60% of total energy consumption (European-Environmental-Agency 2018). Reducing energy usage and increasing efficiency in these two sectors is crucial for achieving the European Union’s 2050 roadmap goals, which require an 80% reduction in overall emissions from 1990 levels. Therefore, the review examines current and potential policy instruments to promote energy sufficiency and conservation, including progressive home appliances, buildings, and vehicle standards. The review also examines the energy crisis and explores the potential of renewable energy sources to address it. It focuses on how energy can be saved in buildings and vehicles, including the use of electric and hydrogen-powered vehicles. In addition, the review investigates the role of artificial intelligence in assisting energy saving initiatives in various industries. Furthermore, case studies of successful energy saving initiatives and technical solutions for energy storage are included in the review. The overall scope of the review encompasses a comprehensive strategy for optimizing energy consumption and lowering emissions in the building and transportation sectors.

## Current status of the energy crisis

Energy is one of the most crucial aspects of modern society, powering economies, transportation, and daily life. Over the years, the world has relied on various energy sources, including coal, oil, natural gas, and renewable sources like wind, solar, and hydro. However, as the global population continues to grow, energy demand has increased, leading to an energy crisis that has far-reaching impacts on the environment, economy, and society. In addition, the Russian invasion of Ukraine in early 2022 abruptly ended the recovery in global energy consumption after the pandemic-caused decline in 2020. This invasion also stoked inflationary pressures and slowed economic growth. Market tensions were present before Russia invaded Ukraine but have been significantly exacerbated. This has caused energy prices to fluctuate and spike sharply, especially for natural gas in European markets, and the threat of further supply disruption looms large.

Renewable energy growth has done well despite this unrest. As a result of the crisis, many nations have had to reevaluate their needs for energy security. The crisis has shattered energy relationships with Russia that were based on the premise of trust and reliable supplies. The energy trade and investment landscape are being profoundly recast due to this. It has already sparked several actions to enhance energy security, such as funding for developing domestic manufacturing capacity in essential industries. One important open question is whether the current crisis will speed up energy transitions or whether a confluence of unstable economic conditions and hurried policy decisions will cause momentum to slacken (IEA 2022b). Record-high fossil fuel prices and rising emissions provide compelling arguments for reducing or eliminating our reliance on these fuels. In addition, concerns about energy security might encourage renewed investments in the infrastructure and supply of fossil fuels. The impact of various policy options is taken into account in this outlook.

In 2021, following the war, the European Union imported over 380 million cubic meters of gas per day, equivalent to around 140 billion cubic meters, from Russia via pipeline. In addition, 15 billion cubic meters of liquefied natural gas were also delivered. These imports from Russia accounted for about 45% of the European Union’s gas imports and nearly 40% of its total gas consumption in 2021 (IEA 2022b). The European Union is confronted with an additional challenge in this unsettling situation. This challenge is the potential disruption of natural gas imports from Russia. As a result of this disruption, the security and affordability of the bloc’s energy supply are seriously threatened, causing Member States to make plans for a sharp decline in gas demand by late 2022 (Wang et al. 2023).

**Fig. 2:**
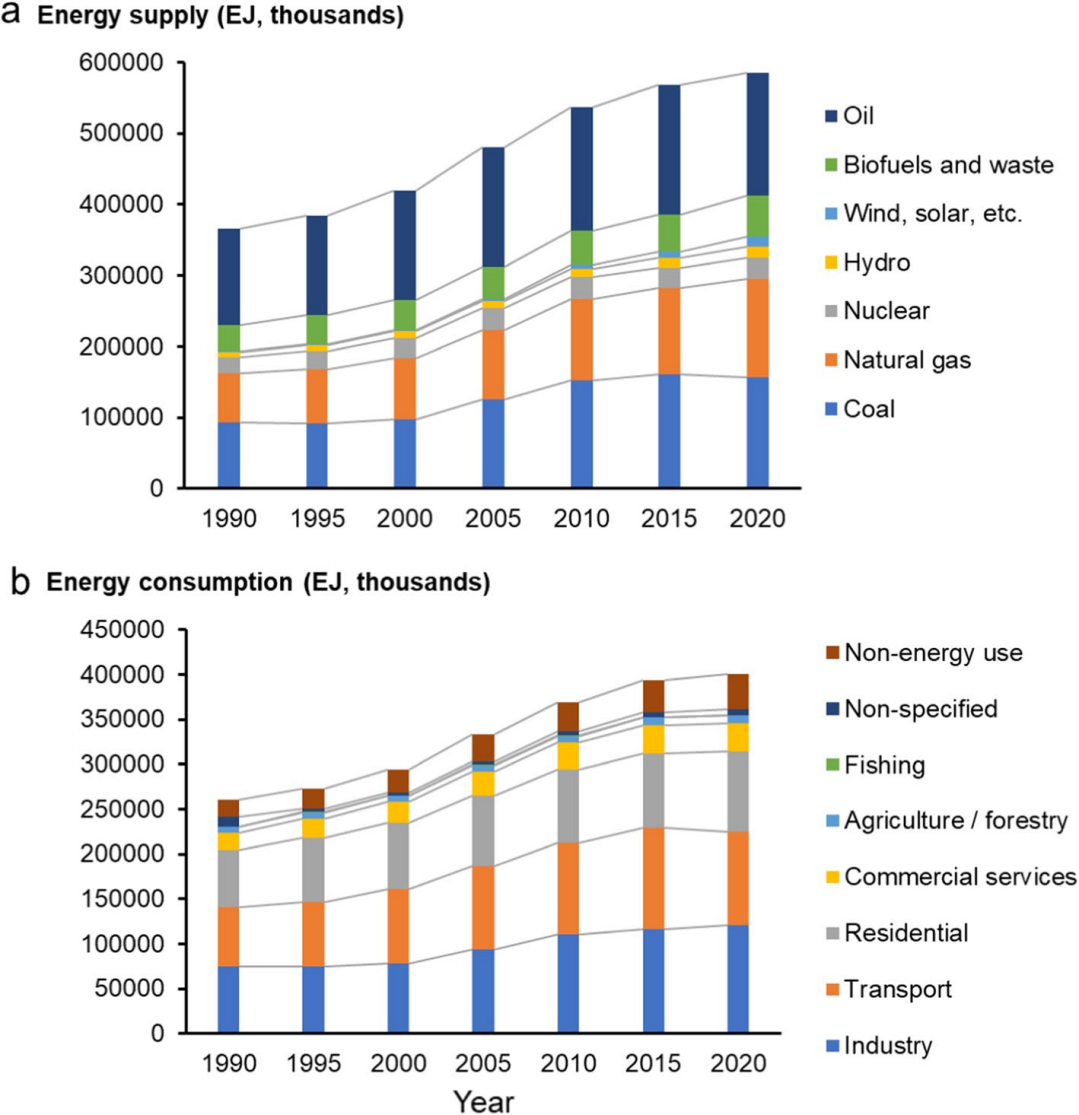
Worldwide energy supply from 1990 to 2020. In 2020, the global energy supply was 584,523,552 EJ, of which 29.47% was derived from oil, 26.80% from coal, 23.68% from natural gas, 9.84% from biofuel, and 5.21% from renewables (**a**). Additionally, energy consumption accounted for 400,819,444 EJ in 2020 (**b**), with 30.01% used by industry, 26.18% by transport, 22.08% by residential, 7.92% by commercial and public services, 2.15% by agriculture, and 1.77% for other uses. *Source*: (IEA 2022c). EJ: exajoules

The prices of many fossil fuels fell to their lowest levels in decades due to the historic decline in global energy consumption in the early months of the COVID-19 crisis in 2020. The price rebounds, however, have been incredibly swift since mid-2021. After briefly falling in 2020, oil prices have since rebounded to around or above $100 per barrel. In 2020, the worldwide energy supply was 584,523,552 EJ (Fig. 2a), while energy consumption accounted for 400,819,444 EJ in 2020 (Fig. 2b). The cost of coal has risen to record levels. On an energy-equivalent basis, spot natural gas prices in Europe have routinely exceeded $50 per million British thermal units, more than twice the price of crude oil. Due to tight gas and coal markets, electricity prices have been unusually high in many markets. The global energy crisis has had a negative impact on individuals, businesses, and entire economies around the world.

Guan et al. (2023) used a global multi-regional input–output database and detailed household expenditure data to model rising energy prices’ direct and indirect impacts on 201 expenditure groups across 116 countries. Through various energy price scenarios, they found that household energy costs would increase by 62.6–112.9%, resulting in a 2.7–4.8% increase in household expenditures. Energy cost burdens varied across household groups due to differences in consumption patterns, supply chain structure, and energy needs. The escalation of living costs may potentially push an additional 78 million to 141 million people into extreme poverty. To alleviate the energy crisis, targeted energy assistance can support vulnerable households with a particular focus on necessities such as food.

The energy crisis, including economic, political, and environmental factors, significantly impacts society. One of the most visible impacts is the high energy cost affecting both developed and developing countries, which has a ripple effect on the economy. For instance, transportation costs have risen due to high fuel prices, leading to higher prices of goods and services. This, in turn, leads to inflation and reduced purchasing power for consumers, thus impacting the economy negatively. Moreover, energy is critical in various industries, including manufacturing, agriculture, and construction. Therefore, the energy cost directly impacts production, reducing business profit margins. This, in turn, can lead to reduced investment, lower growth, and higher unemployment rates.

The energy crisis has had far-reaching impacts on the environment. Where over-reliance on fossil fuels, which are finite resources, has led to increased greenhouse gas emissions, contributing to climate change. The combustion of fossil fuels releases carbon dioxide, methane, and other greenhouse gases, trapping heat in the atmosphere and leading to global warming (Chen et al. 2022; Farghali et al. 2022). Moreover, the extraction and transportation of fossil fuels have also led to environmental degradation. For instance, oil spills have destroyed marine life and ecosystems, while coal mining has resulted in soil erosion, deforestation, and air pollution (Worlanyo and Jiangfeng 2021). The environmental impact of the energy crisis is not limited to fossil fuels but also extends to renewable energy sources. The construction of wind turbines and solar panels can lead to habitat destruction, soil erosion, and pollution, impacting the environment negatively (Osman et al. 2022a).

The energy crisis has had significant social impacts, particularly in developing countries. The lack of access to energy, particularly electricity, has reduced many people’s quality of life (Cantarero 2020). For instance, lack of electricity limits accesses to modern healthcare, education, and communication, hindering social and economic development. Moreover, the energy crisis has also led to social inequality, with marginalized communities often having limited access to energy resources. In some developing countries, people rely on biomass, such as wood and charcoal, for cooking and heating, leading to deforestation and health issues. The energy crisis has also led to the displacement of communities, particularly in areas with high energy demand. For instance, the construction of hydroelectric dams has led to the displacement of indigenous communities in many parts of the world (Osman et al. 2022a). In Africa, people living without electricity increased by more than 15 million (or 3%) between 2019 and 2021, reversing almost all the gains made over the previous five years. This alarming trend is set to be reinforced in 2022 (IEA 2022d).

## Green solutions for home heating

Several green alternative solutions are available for oil or natural gas-based home heating systems that can help reduce carbon emissions and improve energy sustainability. Air and ground source heat pumps are more efficient and sustainable than traditional heating systems. At the same time, biomass boilers burn wood pellets, chips, or logs to provide heat, which can be a sustainable alternative to fossil fuels. However, they require regular maintenance and a larger storage space for energy.

In addition, solar thermal panels use energy from the sun to heat water or air for space heating, which can be a renewable and sustainable alternative to traditional heating systems. Infrared heating panels use infrared radiation to heat objects in the room, providing more targeted and efficient heating with little maintenance. Geothermal heating, which uses the earth’s natural heat to provide heating and cooling, is also a sustainable and efficient alternative to traditional heating systems. Consultation with a professional is recommended to determine the most suitable green heating solution based on location, climate, size of the home, and available renewable energy sources.

### Biomass boilers and stoves

Burning biomass instead of oil and gas in boilers has successfully combat the greenhouse effect and is considered the most feasible energy option for reducing greenhouse gas emissions (Meloni et al. 2019). Central heating systems, such as stoves and boilers, can utilize a forced-air or hydronic distribution system with radiant or convective heat emitters to generate heat in one location and distribute it throughout a home (Wang et al. 2019). Popular biomass fuels for boilers include pellet straw, energy crops, herbaceous fuels, or a mixture. Additionally, pellet fuels with larger surface areas are more easily burned.

Biomass boilers have the potential to promote energy independence, lower heating costs, and reduce greenhouse gas emissions (Osman et al. 2023). Stephen et al. (2016) noted that decentralized wood pellet boilers were successfully implemented in the Bella Coola hamlet, offering economic benefits compared to conventional diesel combustion systems. The simplicity of operation and reduced power needs for hot water heating were key advantages. Las-Heras-Casas et al. (2018) found that replacing traditional fossil fuel and natural gas boilers with biomass boilers reduced carbon dioxide emissions by 94% and saved between 39.24 and 62.71% of energy costs over the entire life cycle, making biomass combustion an economically attractive option. In addition to being a low-carbon heating technology, wood pellet heating can be more cost-effective for those with limited budgets. They can be purchased in small quantities, easing the burden on those with limited incomes. However, biomass heating systems, such as boilers or stoves, have various drawbacks related to necessary space, efficiency, emissions, and maintenance. Compared to boilers powered by fossil fuels, biomass boilers have higher installation and maintenance costs.

Biomass-based low-carbon heating systems can significantly reduce greenhouse gas emissions from residential buildings, as biomass is essentially carbon neutral, resulting in a low carbon footprint (Wang et al. 2018a). For instance, using wood chips and pellets as fuel in biomass boilers can save up to 0.039 Mtons of carbon dioxide emissions for 54,241 homes over 30 years, compared to diesel combustion (Rafique and Williams 2021). In a heating system that used a mixture of 20% straw and 80% coal, Chowdhury et al. (2019) found that increasing the proportion of straw in the mixture reduced nitrous oxide and sulfur dioxide emissions, demonstrating the carbon reduction effect of biomass. However, Pognant et al. (2018) showed that woodchip boilers might not be as environmentally friendly as natural gas boilers locally but can still significantly improve local air quality by reducing ground-level particulate matter concentrations. Thus, biomass boiler heating can be an effective means of reducing greenhouse gas emissions while having the potential to cause pollution from other gases.

Biomass boilers are renowned for their high heating efficiency (Kütt et al. 2018), especially when using solid biomass fuels with 14–23 MJ/kg calorific values (Gravalos et al. 2016). Wood chip pellets powered by 50 kW boilers have peak mass collection efficiencies of around 98%, with overall collection efficiencies ranging from 70 to 90% (Baumgarten et al. 2022; Wang et al. 2017a). The combustion efficiency of biomass wood can be further enhanced by compressing it into pellets under high pressure and temperature (Hartmann and Lenz 2019). However, the susceptibility of biomass combustion to boiler deposits can cause corrosion on heating surfaces, reducing the combustion efficiency (Chen et al. 2021).

In summary, biomass boilers and stoves offer a compelling alternative to conventional oil and gas heating systems due to their lower heating costs and reduced greenhouse gas emissions. Moreover, they boast higher combustion efficiency, providing more heat to the home.

### Hybrid heat pumps

Heat pump systems, which extract heat from the air, water, or earth for space and water heating, require electricity to operate (Meles and Ryan 2022). Heat pump technology can significantly outperform traditional boiler-based household space heating methods. In fact, advancements in heat pump technology and renewable energy sources could substantially reduce greenhouse gas emissions in the residential sector.

Bianco et al. (2017) analyzed Italy’s shift from household gas boilers to electric heat pumps, highlighting the main energy and carbon reductions. The benefits of heat pumps can be further enhanced if the electricity used to run them has low or no carbon emissions. Brockway and Delforge (2018) found that hybrid heat pump water heaters can reduce hot water usage by 50–70% compared to natural gas water heaters, resulting in deep decarbonization and offering multiple benefits for decarbonizing home heating. Joshi and Dhoble (2018) conducted research on photovoltaic heat pumps, which generate electricity and hot water for households in the UK region, reducing annual electricity consumption by 60% and lowering the overall cost compared to traditional heating systems. Moreover, this approach reduces carbon dioxide emissions by 910 kg/year per household. Increasing the number of photovoltaic panels from 12 to 24 in an intermittent photovoltaic heat pump can further reduce annual carbon emissions by 700 kg/year, making it a practical and sustainable greenhouse gas reduction solution for the UK.

Hybrid heat pump systems offer significant improvements in heating efficiency. According to Obalanlege et al. (2020), a hybrid photovoltaic heat pump system can increase total power generation by 3.5%, with a 6.5% increase in photovoltaic panel power in a 100-fold increase in heating water capacity and improved overall heating performance. The heat pump can adjust the proportion of different energy operations based on weather conditions to ensure that it can still output more heat energy during extreme weather conditions. You et al. (2021) demonstrated higher energy savings (53.1%) and reduced carbon dioxide emissions by 52.0% compared to conventional heat pumps, showing improved energy efficiency and environmental benefits.

Utilizing hybrid heat pumps for space heating can result in cost savings on combustion expenses due to their efficient performance, which reduces energy wastage and promotes sustainable economic practices. In a study by Ran et al. (2020), a combination of solar and air source heat pumps was used to efficiently utilize solar energy during defrost conditions and steadily heat buildings while minimizing energy consumption (reducing it by 9–52%). This hybrid system was implemented in Lhasa and Beijing, resulting in significant economic benefits with a maximum annualized cost savings of 23.4%. Similarly, You et al. (2021) demonstrated a cost savings of 56.4% for residential and office applications through the simultaneous generation and recovery of heat economically and straightforwardly. Hybrid heat pump systems that consist of electrically driven air source heat pumps and gas boilers, as demonstrated by D'Ettorre et al. (2019), can also reduce energy costs by 8%, making them an attractive and cost-effective solution.

Despite their advantages, hybrid heat pumps are limited to providing year-round heating for houses (Kieft et al. 2021). Factors such as solar radiation, temperature, and uneven capacity maintenance contribute to this limitation. Additionally, it could impede the rapid advancement of this technology (Biglarian et al. 2019). The complex structure of hybrid heat pumps results in additional installation and maintenance expenses, and further optimization of the economic feasibility assessment of their life cycle is necessary. Developing energy saving and environmentally friendly refrigerants is essential to enable hybrid heat pump systems that regulate more stable temperatures (Mohanraj et al. 2018).

Hybrid heat pumps present a more effective solution for home heating, creating an opportunity to achieve substantial decarbonization and cost savings within the building industry. By incorporating renewable energy sources, hybrid heat pumps can minimize carbon dioxide emissions and improve the efficiency of traditional heat pumps for heating purposes, reducing energy consumption. This decrease in energy consumption subsequently leads to reduced combustion costs, highlighting the economic feasibility of hybrid heat pumps. However, the drawbacks of low capacity, high installation and maintenance expenses, and significant cooling requirements must also be considered.

### Geothermal heating

Geothermal energy is a dependable, robust, and sustainable energy source generated by the earth’s internal heat. It allows users to heat their homes autonomously without relying on conventional energy providers (Bleicher and Gross 2016; Soltani et al. 2019). Geothermal energy systems can be categorized into three primary groups: direct-use and district heating systems, power production, and geothermal heat pumps. Additionally, geothermal systems can provide active and free cooling to fulfill cooling requirements (Esen et al. 2017). Furthermore, economies of scale can be achieved with larger geothermal installations in the network, resulting in higher efficiency and lower unit energy costs for geothermal heating and cooling.

Baba and Chandrasekharam (2022) found that direct geothermal heating of homes and hotels in Turkey utilizes 2339 MWt of heat, resulting in a 40% reduction in carbon dioxide emissions. In Pratiwi and Trutnevyte (2021) study, geothermal energy was combined with waste incineration and natural gas boilers to replace 50% of the natural gas consumption, leading to a significant decrease of 60% in greenhouse gas emissions. The low environmental impact of geothermal energy further increases the installed capacity. It improves the efficiency of heat production, making it a desirable low-carbon option for the building industry in Geneva. Geothermal resources can also address China’s energy crisis, air quality concerns, and carbon emissions, as demonstrated by Zhang et al. (2020), who found that geothermal heating could lower carbon emissions by 76% and fossil fuel usage by 78% in 2017. Additionally, incorporating renewable energy in the heating system can improve the carbon reduction performance of geothermal energy, as seen in the case of photovoltaic–geothermal heating in the Batna province. The combination of renewable energy in the heating system can further enhance the geothermal carbon reduction performance; the use of photovoltaic–geothermal heating in the province of Batna saves about 15.21 × 10^6^ MWh of energy and 19.17 × 10^5^ tons of carbon emissions per year, contributing 2.28% to the global carbon reduction (Aksas et al. 2015).

Neves et al. (2020) demonstrated that geothermal systems could replace conventional heating and cooling systems, resulting in a 27% reduction in natural gas and electricity heating energy consumption. The high performance and efficiency of geothermal systems lead to significant energy savings and attract customer investment. Similarly, Zyvith et al. (2018) showed that geothermal heating in Massachusetts buildings resulted in cost savings of $1,218,626 (60%), supporting the development of a green economy. Reddy et al. (2020) compared the environmental costs of geothermal heating to conventional heating at the University of Illinois. They found that geothermal heating had significant sustainable economic advantages in all environmental impact categories, while conventional heating systems incurred higher indirect costs. Moreover, geothermal heating systems have up to 50 years of life, offering long-term reliability and durability (Aggarwal et al. 2020).

Geothermal heating has been identified as an economically and environmentally advantageous method of heating homes (Kumar 2021; Chavot et al. 2019). However, the initial investment required for geothermal heat pumps is higher than that for conventional systems, which can impede their development (Chahartaghi et al. 2019). High installation costs, infrastructure requirements, and system designs limit the adoption of geothermal systems (Chen et al. 2019). To address this, Ikeda et al. (2017) used a numerical simulation model with dynamic ground temperature assumptions to optimize the operational costs of a hybrid geothermal heating system, achieving up to 12.56% savings in operational costs. Similarly, Farzanehkhameneh et al. (2020) utilized evolutionary algorithms to optimize the length, number, and radius of wells, external pipes, and in-pipe flow rates, resulting in improved thermal performance and reduced starting costs.

To summarize, geothermal energy is a sustainable and efficient way to meet long-term thermal energy needs and provides a low-carbon renewable option. It has the potential to reduce carbon dioxide emissions significantly, energy demand, and mitigate global warming. However, the expensive upfront investment in geothermal heating systems limits their development.

### Solar photovoltaic systems into electric boilers

Electric boilers are cost-effective for low-cost electricity (Johansson and Göransson 2020). An electric boiler connected to a solar system through an alternating current is called a photovoltaic-assisted electric boiler. To ensure optimal performance, the electric boiler is sized to match the photovoltaic system’s peak alternating current power output (McMillan et al. 2021). When implementing a strategy for using photovoltaic-driven electric boilers, the size of the photovoltaic panels must be considered.

Akhtari et al. (2020) demonstrated that combining photovoltaic, hydrogen, and wind energy could generate electricity for combustion cell heating, with excess electricity fed into an electric boiler. This approach resulted in a 4.5% increase in renewable energy share and a 48% reduction in carbon emissions. Yu et al. (2018) found that integrating solar cogeneration with electric boilers can reduce the waste of excess thermal energy generated by photovoltaics by channeling it to electric boilers for conversion into thermal energy. Electric boilers offer a way to use excess electricity to meet thermal loads, while photovoltaics provide a renewable energy source for significant carbon and cost savings. However, Fitó et al. (2021) found that the energy efficiency of photovoltaic-electric boilers in residential heating technologies is only 20%, which is the worst compared to other energy combination strategies. Nonetheless, the energy cost of the photovoltaic-electric boiler is only €0.144/kWh, allowing excess electricity to be sold to the national grid. The system has a high-performance factor, and free solar energy leads to economic balance.

Large photovoltaic systems can make the design of buildings with electric boilers more cost-effective (Becchio et al. 2015). Das et al. (2021) have demonstrated that using an electric boiler in a renewable power system that includes photovoltaics leads to a 29% reduction in annualized costs and a 7% reduction in energy costs, as well as a significant decrease in annual carbon dioxide emissions (49.3%, equivalent to 21,990 kg/year). By integrating a photovoltaic-electric boiler heating system, Chen et al. (2022b) achieved a 58.9% cost saving in life cycle specific ambient heating costs ($2.77/kWh). To replace bulk coal and achieve clean rural domestic heating, Zhang and Gao (2019) combined distributed photovoltaic with a thermal storage electric boiler to generate electricity for rural heating in Beijing. They found that rooftop photovoltaic heat generation can meet the requirements of the electric thermal storage boiler, saving 3.596 tons of greenhouse gas emissions per year and $825.477 in environmental management costs. However, the high operating cost of hybrid solar electric boiler systems remains a significant barrier to their widespread adoption. Further heating efficiency improvements are needed to reduce operating costs.

In summary, integrating photovoltaic systems with electric boilers offers a viable low-carbon heating strategy. By combining renewable energy sources like photovoltaic with electric boilers, excess energy generation can be utilized, leading to higher energy efficiency and a reduction in carbon dioxide emissions compared to conventional electric boiler heating. Additionally, photovoltaic-electric boilers can be cost-effective, offering significant cost savings in energy and annualized costs.

### Solar thermal heating

Solar heating is a promising option for low-energy homes, as renewable solar energy systems can provide various necessities, such as power, hot water, space heating, and even cooling. The underlying principles behind solar systems, particularly solar heaters, are simple yet effective. These include converting the sun’s electromagnetic photons into thermal energy and transmitting it to water for use in homes or industrial activities (Mabrouki et al. 2022). Solar heating offers several benefits, including lower fuel consumption, reduced pollution, and minimal maintenance requirements (Cristofari et al. 2015).

Mohammed and Hamakhan (2021) demonstrated that using solar hot water systems in three regions of Iraqi Kurdistan can reduce carbon dioxide emissions by 771–824 kg per year, indicating the carbon-saving potential of solar heating in reducing fossil fuel consumption. (Hansen and Vad Mathiesen 2018) also investigated the impact of solar thermal energy on carbon dioxide emissions from technology and fuel substitution. Solar heating has been found to have a higher impact on carbon savings during summer (86.8%) due to the higher solar ratio. In contrast, the efficiency of solar heating systems is positively correlated with temperature, although the collector effect is lower in winter, according to Mohammed and Hamakhan (2021). Installing solar thermal energy systems can significantly reduce carbon emissions and contribute to a low-carbon economy.

Solar thermal integration can reduce the overall use of biomass and thus decrease dependence on this highly demanded energy source in future. In temperate areas, Gauer and Pahn (2022) utilized multifunctional building components to store solar thermal energy as part of a heating system for single-family homes, resulting in a 25% reduction in electrical energy consumption and increased thermal comfort. Combining solar thermal with other renewable technologies can further improve performance, as demonstrated by the integration of solar heating systems with air heat exchangers, which effectively reduced carbon emissions (21.1 tons) and energy demand (37.9 €) during heating (Benzaama et al. 2021). In Sweden, solar thermal energy was integrated into an elderly home, achieving 100% renewable heating and meeting 37% of the thermal energy demand without seasonal storage devices, demonstrating excellent heating performance (Joly et al. 2017). Therefore, solar heating is a highly efficient heating technology.

In conclusion, solar heating is a sustainable and eco-friendly technology that helps to decrease the reliance on non-renewable fossil fuels and subsequently lowers greenhouse gas emissions. Furthermore, integrating solar and thermal energy reduces the need for electricity generation, leading to energy efficiency and cost savings.

### Compressed natural gas and methane

Methane, the primary component of natural gas, has a low carbon-to-hydrogen ratio, resulting in lower emissions of carbon and particulates (Zhang et al. 2022). Compressed methane has a specific volume of around 0.0008 m^3^/kg (Chowdhury et al. 2016). Compressed natural gas is created by compressing regular natural gas to less than 1% of its volume at normal atmospheric pressure and has a high heat of combustion of 47.5 MJ/kg. It is held in a rigid container at 2900–3600 psi (Khan et al. 2015). Biogas can be concentrated to the same level as natural gas by biogas upgrading and distributed through home pipe systems. It can be used for combined heat and power generation, home cooking, and heating (Kadam and Panwar 2017; Ding et al. 2013). Kovalev et al. (2021) explain compressing landfill gas by cooling and compressing the biogas/landfill gas with water to produce a mixture of natural gas hydrate transported to the enterprise for further processing. Biomethane can also be compressed to the necessary pressure and injected into the natural gas pipeline network through a city gate station (Hoo et al. 2018),

Natural gas with a high-octane rating possesses desirable characteristics such as clean combustion, high calorific value, and abundant reserves. It also has a high hydrogen/carbon ratio and broad combustion limits (Szwaja et al. 2018). Therefore, the compression and utilization of landfill gas are highly competitive with methane alone (Kokabi et al. 2021). Compared to gasoline, compressed natural gas emits 10–13% less carbon dioxide and 15–30% less carbon monoxide while having approximately 25% lower combustion level than gasoline, which enhances fuel combustion performance (Lather and Das 2019). Using compressed natural gas to supplement diesel has the potential to reduce nitrogen oxide and particulate matter emissions while improving fuel efficiency, as found by Langness et al. (2017). By adding compressed natural gas, conventional fuel combustion power can be increased. For instance, adding 56% compressed natural gas to diesel fuel can increase the maximum power rating by 30% (Bogdanov and Dimitrov 2019). Methane from biomass sources can meet all or part of the compressed natural gas demand, fueling internal combustion engines at higher compression ratios to pursue higher efficiencies (Zhou et al. 2019).

Furthermore, compressing gas streams rich in methane to 70 bar for transportation via pipelines can recover up to 86.4% of the carbon in biomass in the form of methane. Therefore, compressed natural gas can be utilized in generators that use conventional fuels, offering both economic advantages in terms of fuel savings and environmental advantages in terms of carbon savings. However, the combustion of compressed natural gas may lead to incomplete combustion due to slow combustion rates, resulting in the emission of unused methane into the atmosphere, which could potentially cause a 20-fold increase in global warming potential (Rahman and Ahmad 2019). In summary, using compressed natural gas and methane improves fuel combustion efficiency, making it economically viable while reducing greenhouse gas emissions and promoting cleaner energy.

**Fig. 3:**
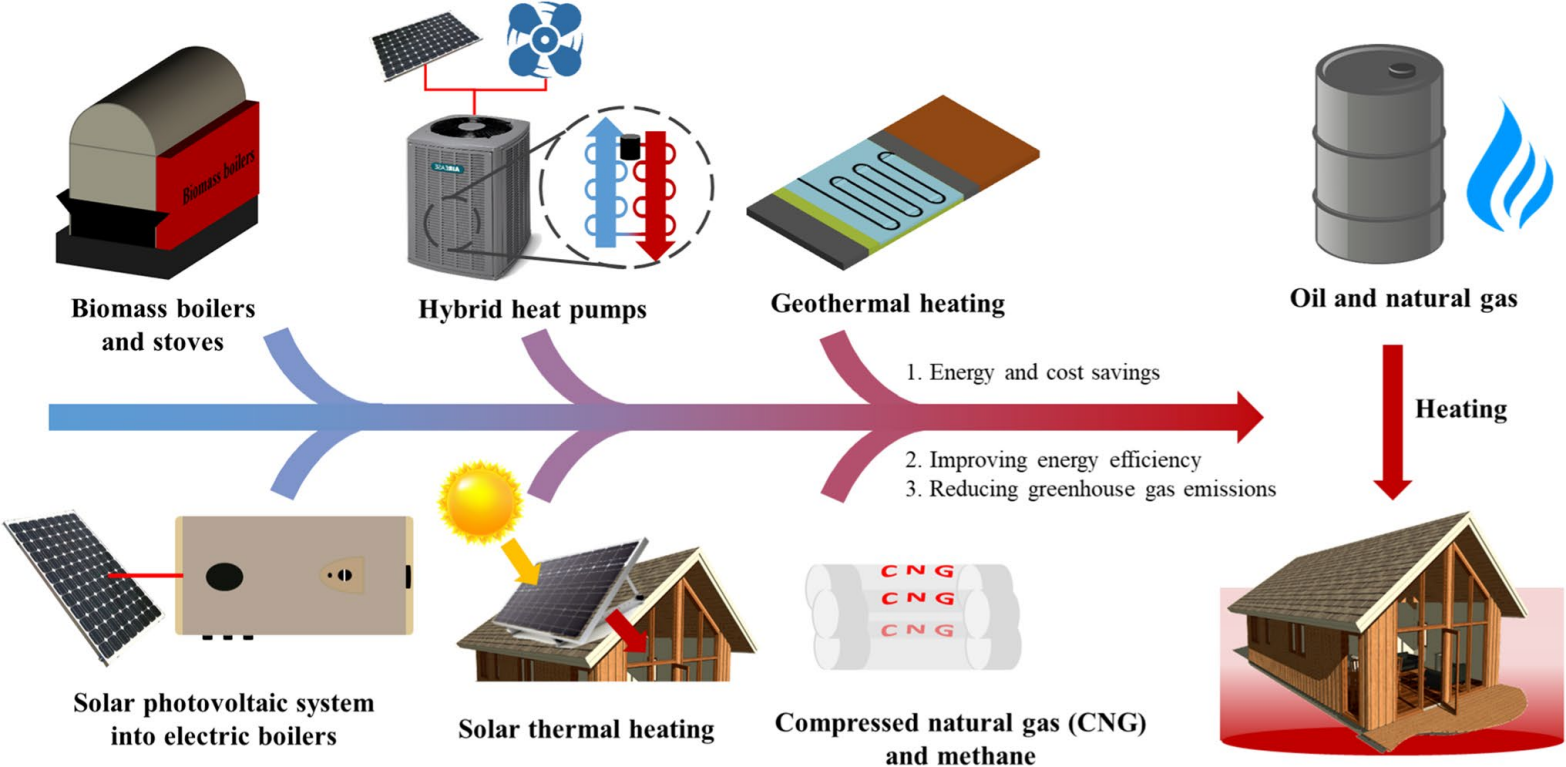
Strategies to replace oil and natural gas heating. These include biomass boilers and furnaces, hybrid heat pumps, geothermal heating, solar photovoltaic systems converted into electric boilers, solar heating, and compressed natural gas and methane. By adopting these green alternative energy solutions, it is possible to significantly reduce energy consumption and costs while achieving efficient heating. Furthermore, these solutions increase the efficiency of energy combustion, resulting in enhanced heat production and reduced greenhouse gas emissions

This section presents various alternative energy solutions to replace oil and natural gas heating, including biomass boilers and stoves, hybrid heat pumps, geothermal heating, solar photovoltaic systems integrated with electric boilers, solar thermal heating, and compressed natural gas and methane, as shown in Fig. 3. These strategies effectively enhance the energy efficiency, cut costs, and reduce carbon dioxide emissions.

## Energy saving in buildings

The building sector is responsible for significant energy consumption and carbon emissions, making it an important area for emissions reduction (Chen et al. 2022a; Liu et al. 2022). According to the International Energy Agency, the building sector is projected to account for 34% of global final energy consumption in 2021, with direct and indirect emissions from the construction sector contributing to 33% of global energy emissions (International Energy Agency 2022). Moreover, a United Nations Environment Programme report suggests that direct carbon emissions from the building sector must be reduced by at least 50% by 2030 to achieve a net-zero-carbon building stock by 2050 (United Nations Environmental Programme 2020). Building operations contribute 30% of total global energy consumption and 27% of total carbon emissions from the energy sector over the building life cycle (Daioglou et al. 2022), with energy consumption vary by building type, such as residential, commercial, and large buildings (Almushaikah and Almasri 2021; Chou and Ngo 2016). According to Chen et al. (2023), the construction industry is a significant consumer of non-renewable energy and a substantial contributor to greenhouse gas emissions. Construction activities are responsible for 36% of global energy consumption and 39% of global carbon dioxide emissions. The authors analyzed carbon emissions throughout the building’s life cycle and found that the construction phase contributes between 20 and 50% of total carbon emissions. Table 1 summarizes energy saving measures and outcomes for residential, commercial, and large buildings in various countries and regions. This is to meet the Paris Agreement targets and the European Commission’s goal of zero-carbon buildings for all new buildings from 2030 onwards, as well as retrofitting existing buildings to zero-carbon buildings by 2050 (European Commission 2022).

**Table 1:** Strategies for achieving energy saving efficiency in buildings in different countries and regions. Strategies include optimizing passive design, implementing energy-efficient building systems, and developing intelligent energy consumption monitoring and analysis systems, depending on the type of building

Country	Year	Building type	Energy saving strategies	Critical findings	References
Kingdom of Saudi Arabia	2021	Residential buildings	Solar energy	The analysis focuses on two regions in the Kingdom of Saudi Arabia where two energy measures reduced energy consumption by 33% and 32%, respectively. However, the region’s adoption of solar energy on residential rooftops has not been widely promoted, and its economic feasibility requires further evaluation	Almushaikah and Almasri (2021)
			Highly efficient heating, ventilation, and air conditioning systems		
China	2016	Residential buildings	Smart grid big data analytics framework	The proposed framework suggests an intelligent approach to energy saving by regulating the operating hours of household appliances. However, the framework is currently in the conceptual stage, and its practical implementation in residential buildings is yet to be determined. Additionally, the accuracy of its energy analysis requires further evaluation	Chou and Ngo (2016)
			Energy saving decision-support system		
Iran	2020	Residential buildings	Light shelves	The study presents a conceptual design that utilizes light shelves to reduce a building’s total electrical load demand by 5–10% in various scenarios, resulting in energy savings. Nonetheless, the practical application of this design in architectural housing requires further investigation and analysis	Ebrahimi-Moghadam et al. (2020)
Iran	2017	Residential buildings	Green roof	Passive energy saving techniques like green roofs, roof ponds, wind catchers, and underground houses have reduced building energy loss. However, their application is limited by certain factors. For example, underground houses built only one meter below the surface may not effectively save building energy	Goudarzi and Mostafaeipour (2017)
			Roof pond		
			Wind catcher		
			Underground house		
Singapore	2017	Commercial buildings	Optimization of multi-chiller plant	The study focused on optimizing multiple chillers in buildings to reduce energy consumption and demonstrated that chillers of varying sizes could reduce energy consumption in commercial buildings by 20–40%. However, it was noted that multiple chillers might be more beneficial in tropical areas to reduce cooling load losses, whereas, in colder regions, they may need to be optimized differently	Thangavelu et al. (2017)
USA	2016	Commercial buildings	Material selection	The study examines a range of passive design strategies and energy-efficient building systems that can be optimized to reduce energy consumption in commercial buildings to achieve net-zero energy targets. However, the economic feasibility of implementing these strategies is still being evaluated, and the current analysis phase is experimental	Aksamija (2016)
			Improvements to the building envelope		
			Retrofitting of heating, ventilation, and air conditioning systems		
			Retrofitting lighting systems		
			Retrofitting occupancy loads		
			Application of renewable energy sources		
Singapore	2019	Commercial buildings	Smart-token-based scheduling algorithm	The study proposes using the Internet of Things technology in commercial buildings' heating, ventilation, and air conditioning systems to reduce energy consumption. Case studies demonstrate a potential energy reduction of up to 20%. However, further investigation is necessary to determine its applicability in other building types	Png et al. (2019)
Qatar	2021	Commercial buildings	Retrofitting of heating, ventilation, and air conditioning systems	The study aimed to reduce energy consumption in Qatar’s 7000 m^2^ office building by optimizing its heating, ventilation, and air conditioning systems and loads. The study results showed that the optimization significantly saved building energy consumption. However, the economic analysis revealed that the payback period for the optimization measures is relatively long	Alns and Sleiti (2021)
			Retrofitting occupancy loads		

These measures are intended to decrease energy consumption and enhance building efficiency. In addition, the table provides examples of energy saving cases in residential and commercial buildings, which can serve as a reference for similar projects

Table 1 illustrates the energy saving measures employed in different countries and regions for various building types. The primary focus is optimizing passive design strategies, building energy-efficient systems, and developing intelligent energy monitoring and analysis systems to reduce energy consumption. Four case studies demonstrate different strategies used for residential buildings from 2016 to 2021 in China, Iran, and the Kingdom of Saudi Arabia. China employs two strategies: the smart grid big data analytics framework and the energy saving decision-support system. Iran uses five techniques: green roofs, roof ponds, wind catchers, underground houses, and light shelves.

On the other hand, the Kingdom of Saudi Arabia uses two strategies, namely solar energy and highly efficient heating, ventilation, and air conditioning systems from China and Iran (Chua et al. 2013). As heating, ventilation, and air conditioning systems account for most energy consumption in buildings, they are considered one of the best measures to reduce energy consumption. However, the economic viability of these measures needs further analysis.

Furthermore, as shown in Table 1, the four residential building energy saving strategies effectively reduce energy consumption, with the Kingdom of Saudi Arabia achieving up to a 32% reduction (Almushaikah and Almasri 2021). However, the promotion and economic evaluation of solar energy in some parts of the Kingdom of Saudi Arabia still require further exploration (Tlili 2015). The table also highlights the potential of the smart energy efficiency decision framework to save energy by scheduling the operating hours of appliances. Still, its application in residential buildings and the accuracy of its energy analysis remain to be determined (Chou and Ngo 2016). Passive strategies, such as light shelves, green roofs, roof ponds, wind catchers, and underground houses, can also reduce energy losses in building maintenance structures. Still, engineering limitations exist, such as the ineffectiveness of underground houses built one meter underground in saving energy in buildings (Ebrahimi-Moghadam et al. 2020; Goudarzi and Mostafaeipour 2017).

For commercial buildings, Table 1 analyses various strategies used to reduce energy consumption in commercial buildings across the USA, Singapore, and Qatar from 2016 to 2021. Six energy saving strategies were employed in the USA, including material selection, building envelope improvements, heating, ventilation, and air conditioning systems, retrofitting lighting systems, retrofitting occupancy loads, and application of renewable energy sources. While these passive design strategies and optimized energy-efficient building systems can effectively achieve net-zero energy goals, their economic costs require further analysis as they are currently in the experimental phase (Aksamija 2016). Additionally, two case studies in Singapore demonstrate the effectiveness of optimizing multi-chiller plants and smart-token-based scheduling algorithms in reducing building energy consumption. However, these strategies may not achieve energy savings in other regions due to differences in building types and regional influences (Png et al. 2019; Thangavelu et al. 2017).

Additionally, Table 1 presents an analysis of a 7000-square-meter office building in Qatar where the optimization of heating, ventilation, and air conditioning systems and loads was implemented to reduce the building’s energy consumption. However, the study found that the payback period for these strategies was relatively long (Alns and Sleiti 2021). Therefore, selecting appropriate strategies for reducing or saving energy in buildings will depend on the building type, country, and region. Strategies such as optimizing passive design, energy-efficient building systems, and developing intelligent energy monitoring and analysis systems can be effective for achieving energy saving goals. Optimizing heating, ventilation, and air conditioning systems is one of the most effective measures for reducing energy consumption in buildings (Aram et al. 2022; Taheri et al. 2022; Yao and Shekhar 2021).

In this section, we examine the various strategies used by residential and commercial buildings across different countries and regions to effectively reduce or save energy consumption in buildings. Our analysis highlights that adopting passive design strategies, energy-efficient building systems, and developing intelligent energy monitoring and analysis systems are practical approaches to achieving energy savings in buildings. Additionally, optimizing heating, ventilation, and air conditioning systems is one of the most effective measures currently available to reduce energy consumption in buildings. However, it is crucial to select appropriate strategies for each building type, depending on the country and region where it is located. To facilitate the early achievement of net-zero energy and the Paris Agreement goals in buildings, conducting a thorough economic evaluation of these strategies and studying their engineering applications is vital.

## Applications of renewable energy sources for energy saving

The rapid depletion of energy supplies, environmental concerns, growing energy prices, and rising energy consumption have all drawn attention to energy saving in recent years (Allouhi et al. 2015; Bah and Saari 2020; Shahsavari and Akbari 2018). Urbanization and industrialization increased energy use and air pollution problems, where cities account for approximately 70% of global energy consumption and 71% of carbon emissions (Lin and Zhu 2019). Therefore, achieving energy sustainability in cities requires increasing energy saving concepts (Martos et al. 2016), growing cities’ energy efficiency through innovative city development (Cai et al. 2019), and applying a carbon reduction strategy (Sodiq et al. 2019). The low-voltage distribution system in buildings (Sodiq et al. 2019) and public lighting systems (Ruparathna et al. 2016) have recently received much attention as potential energy savings options. Since building energy consumption is constantly rising, particularly in industrialized nations, deploying energy saving in structures is crucial. This rise in energy consumption also exceeds energy use in the industrial and transportation sectors (Pérez-Lombard et al. 2008; Sadeghian et al. 2021). According to the International Energy Agency, energy in the building sector accounts for 36% of worldwide final energy use (IEA 2019); hence transition and energy conservation in buildings is crucial to lowering energy and greenhouse gas emissions.

Many efforts have been made to replace the energy consumed by buildings with renewable energy, which increased globally (IEA 2022b) by installing various renewable energy systems, such as photovoltaic systems, geothermal heat pumps, and solar-assisted heat pumps in buildings (Hong et al. 2017; Jeong et al. 2018; Koo et al. 2018). As a result, on-site renewable energy production on buildings has rapidly increased to achieve the energy transition and create net-zero energy buildings (IEA 2022b; Kong et al. 2020; Marszal et al. 2012). In addition, several nations have already implemented laws requiring the installation of renewable energy systems in new construction. For instance, in the USA, building owners must install a 1 kW photovoltaic system for every 10,000 square feet of new commercial or residential space. Furthermore, by 2025, all new federal buildings in Canada will be powered entirely by 100% clean electricity according to the Canadian greening government strategy (Kong et al. 2020). In Spain and Scotland, 10–12% of the total power consumed in new and renovated buildings larger than 1000 m^2^ should come from on-site renewable energy sources (Kong et al. 2020). Following South Korea’s mandatory installation for public building policy, public buildings larger than 1000 m^2^ must supply 30% of their operating energy from on-site renewable energy sources (IEA 2018).

Numerous studies have investigated the feasibility of switching from conventional to clean energy sources following these national policies (Buonomano et al. 2016; Madessa 2015; Tripathy et al. 2017). Some earlier studies investigated using integrated photovoltaic building materials to use self-generated energy as the building’s operating energy (Buonomano et al. 2016; Tripathy et al. 2017). Additionally, research has been done on reducing building energy use for heating and cooling using underground heat sources (Kong et al. 2020; Niu et al. 2015).

## Energy saving in transportation and energy systems 

### Vehicle

The European Union heavily depends on the building and transportation industries, which accounted for over 60% of total energy usage, or about 652.1 million tons of oil equivalent, in 2016 (European-Environmental-Agency 2018). This makes improving efficiency and reducing energy consumption in these sectors crucial for achieving the European Union’s goals outlined in the 2050 roadmap. The roadmap calls for an overall emissions reduction of 80% from 1990 levels by 2050. The building and transportation sectors must reduce their equivalent emissions to 90% and 60% of 1990 levels, respectively (Cao et al. 2017). This highlights the urgent need for measures to be taken in these industries to curb emissions and combat climate change.

**Fig. 4:**
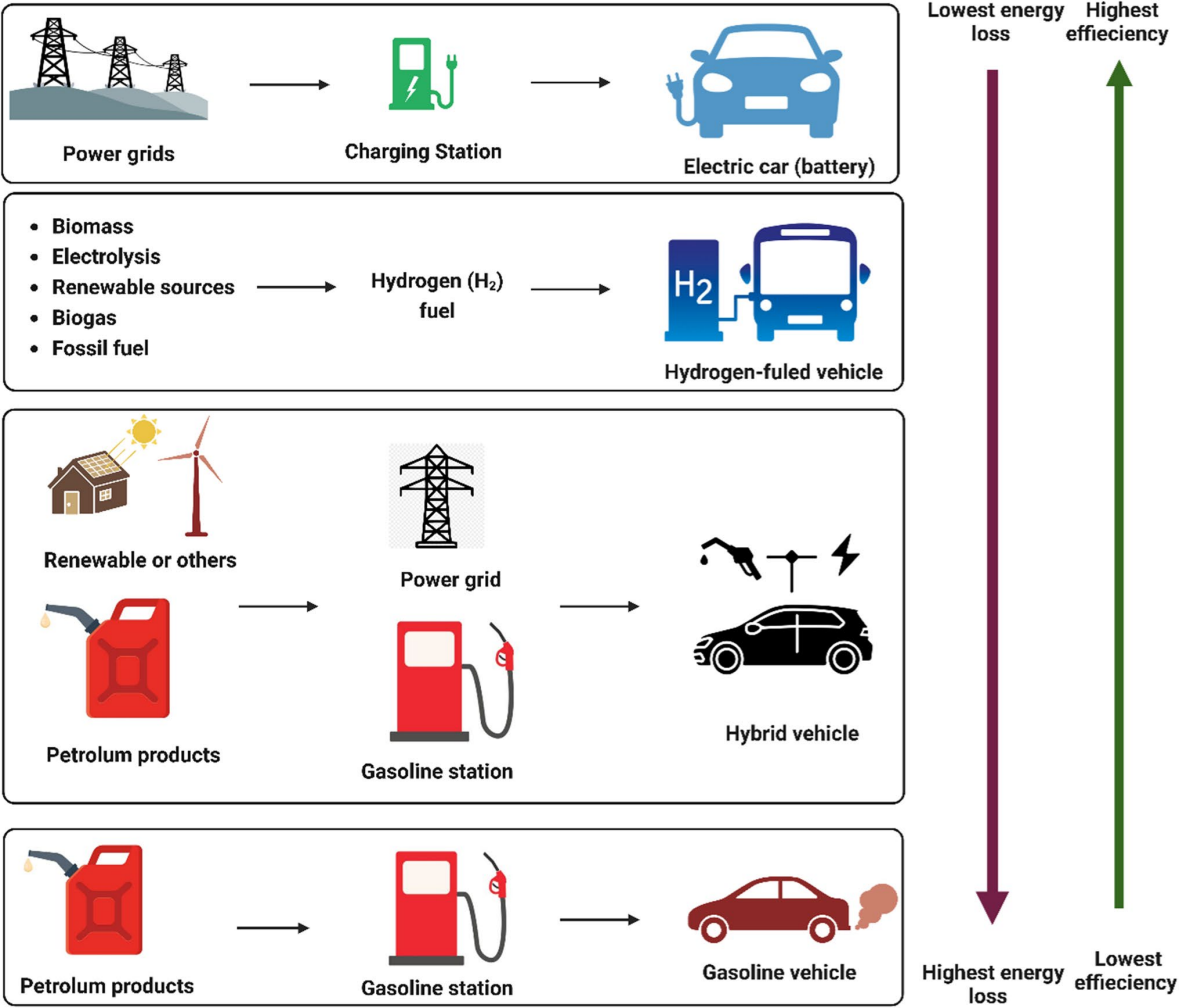
Energy saving potential of different types of vehicles The greatest potential for energy savings and minimal energy loss was demonstrated by electric vehicles, whereas gasoline-powered vehicles demonstrated the opposite. Therefore, electric vehicles can potentially reduce the energy consumption and greenhouse gas emissions of the automotive industry. However, battery-related issues such as limited driving range, extended charging time, high battery costs, and heavyweights must be resolved for optimal performance

Hybrid electric vehicles have seen significant development due to the benefits of both internal combustion engines and battery electric vehicles. By incorporating batteries and electric motors with internal combustion engines, hybrid electric vehicles can improve fuel efficiency and lower greenhouse gas emissions, as shown in Fig. 4. This allows for the implementation of energy harvesting techniques suitable for both internal combustion engine vehicles and battery electric vehicles. As a result, hybrid electric vehicles can generate more energy while emitting fewer greenhouse gases. Bai and Liu (2021) comprehensively review energy harvesting techniques from four perspectives: waste heat recovery from exhaust gas, mechanical energy recovery from braking, vibration, and shock, alternative fuels, and renewable energy integration. The study focuses on several technologies, including thermoelectric generators, the organic Rankine cycle, regenerative shock absorbers, regenerative braking, and solar roofs. After analyzing these technologies, the authors suggest that waste thermal energy recovery is the most promising energy saving approach. Waste heat can be converted into electrical energy using thermoelectric generators, valuable functions using the Rankine cycle, or electrical and mechanical energy using turbo-compounds (Bai and Liu 2021).

Different types of vehicles have varying cost-per-kilometer reductions, with gasoline having 0%, ethanol (E85) having 1%, hybrids having 20%, diesel having 23%, biodiesel having 27%, liquefied petroleum gas (LPG) having 33%, natural gas vehicles (NGV) having 75%, and electric vehicles having the highest reduction of 75% (Yadlapalli et al. 2022). Table 2 and Fig. 4 show that electric vehicles also have the lowest energy loss of 33%, compared to other vehicles such as diesel oil, liquefied petroleum gas, natural gas vehicles, plug-in hybrid vehicles, and fuel cell vehicles, with energy losses ranging from 55 to 84%. However, electric vehicles face significant battery-related challenges, such as limited driving range, long charging time, high battery cost, and heavyweight. The bulky and heavy battery packs take up considerable space inside the car, with an estimated weight of 200 kg, depending on the battery capacity. Therefore, electrical and mechanical components must be lightweight to maintain the vehicle’s weight balance (Yadlapalli et al. 2022). Fortunately, advancements in electric vehicle technology have made a smooth transition from conventional gasoline or diesel-powered vehicles possible.

**Table 2:** Comparative approach at different vehicle types on-road use (Rimpas et al. 2022; Yadlapalli et al. 2022)

Technology	Energy loss (%)	Efficiency (%)	Energy used (kWh/km)
Electric/battery vehicles	33	67	0.28
Fuel cell vehicle	78	22	0.87
Plug-in hybrid vehicles	55	45	0.42
Natural gas vehicle	81	19	1.00
Liquefied petroleum gas	84	16	1.19
Diesel	80	20	0.95
Petrol	86	14	1.36

Electric vehicles have the lowest energy loss compared to other vehicle types, such as diesel oil, liquefied petroleum gas, natural gas, plug-in hybrid vehicles, and fuel cell vehicles. However, electric vehicles face significant battery-related obstacles, including a limited driving range, a lengthy charging time, a high battery cost, and a heavy weight

In recent years, sales of electric/battery vehicles have surged, despite being more expensive than their internal combustion engine counterparts. Only 10 countries account for 95% of all-electric vehicle sales, with China being the largest market. It is predicted that 20 million electric/battery vehicles and plug-in hybrid cars will be sold by 2030, with an estimated 27.5 million sales by 2035 (Yadlapalli et al. 2022). The global market for electric/battery vehicles is projected to quadruple between 2020 and 2026, reaching a market size of approximately 725 billion US dollars by 2026 (Yadlapalli et al. 2022). Furthermore, several countries are preparing for a shift in mobility by restricting the use of fossil fuel-powered vehicles and promoting the adoption of electric/battery vehicles.

Automated driving technology offers energy savings by allowing cars to adjust their motion before future events. According to Vahidi and Sciarretta (2018), connectivity with other vehicles and infrastructure can enable drivers to anticipate potential hazards, such as hills, bends, slow traffic, traffic signals, and nearby cars. Automated and networked vehicles can save between 20 and 30% of fuel by anticipating and collaborating while driving. Several road experiments have demonstrated the technology’s viability, with the potential for 5–15% energy savings in recognized research programs in the USA, Europe, and Japan (Tsugawa et al. 2016; Vahidi and Sciarretta 2018). Static road information, like the road grade, could result in a 3% energy savings from highway driving based on conservative estimates from published experimental findings. In arterial driving, traffic signal data through vehicle-to-infrastructure communication could reduce energy consumption by up to 10%. Reservation-based crossings could save up to 20% when connected and automated vehicles are fully implemented (Vahidi and Sciarretta 2018).

Smart charging of electric vehicles in buildings has received much interest in research projects, as it presents an opportunity to take advantage of their potential and the fact that they require electricity for daily use. Researchers have developed strategies to enhance the energy label of residential buildings by combining solar power systems and electric vehicles, resulting in a reduction of 2.52% in primary energy (Oskouei et al. 2020). Researchers in a lab setting at the University of California have integrated fuel cells, electric vehicles, and battery storage for energy management, saving $3.70 per day (Wang et al. 2016). These studies have demonstrated the economic efficiency of electric vehicles.

Furthermore, the integration of photovoltaics with electric vehicles has been assessed for a two-story residential building in Orlando, USA, resulting in 66% annual overall savings (Alirezaei et al. 2016). In contrast, a university building in Valladolid, Spain, has been investigated for integrating photovoltaics, biomass boilers, and geothermal systems, resulting in 45–52% annual primary savings. Based on the estimated energy savings for the methods mentioned above, all of them have been successful from an energy saving perspective, with the strategy by Kosonen and Keskisaari (2020) having a negative cost.

### Uses of hydrogen as vehicle-fuel

Hydrogen-based renewable energy is increasingly considered one of the most environmentally friendly and promising alternatives to meet the growing energy needs. It is regarded as a clean and efficient energy carrier, making a hydrogen-based economy a potential solution for future energy security and sustainability (Osman et al. 2022b). Although some hydrogen-producing technologies are still in the research phase, they are already commercially available (Sazali 2020). Moreover, hydrogen-rich streams can produce high-value compounds like ethanol, methanol, and gasoline and directly use hydrogen in fuel cells.

The production and use of hydrogen are essential to achieving both environmental and energy goals, and sustainable hydrogen production processes must be a priority for global implementation. According to Maggio et al. (2019), intermediate industrial commodities such as chemical synthesis could emerge as the first green hydrogen market, followed by stationary power generation, which plays a significant role in the transportation industry. Sgobbi et al. (2016) emphasized the crucial role of hydrogen in decarbonizing the European energy system, highlighting its potential, particularly in the transportation sector and industries.

European Union policymakers advocate adopting battery and fuel cell electric vehicles and expanding charging and hydrogen refueling infrastructure to decarbonize road transportation. However, according to Förster et al. (2023) grid-connected hybrid microgrids with on-site hydrogen production, battery and hydrogen energy storage, and renewable energy may not currently be cost-effective for decarbonizing road transportation in a real-world case study in Germany. The authors suggest that amending German demand charge regulations may be necessary to support the sustainable design and operation of upcoming charging and hydrogen refueling microgrids.

Hydrogen’s potential as an energy storage medium is increasingly recognized, and various studies have explored its applications in different sectors. In the heating industry, Samsatli and Samsatli (2015) found that using hydrogen as inter-seasonal storage could help decarbonize the industry in the UK. They found that an 80% electricity and 20% hydrogen mix was optimal for heat production, underscoring the importance of a reliable hydrogen supply network to support carbon capture and utilization technologies.

Meanwhile, Colbertaldo et al. (2019) investigated the potential of hydrogen, produced through the power-to-gas method, for decarbonizing power generation in California using 100% renewable electricity. Their study aimed to reduce greenhouse gas emissions. They evaluated the impact of a novel onboard cold energy utilization system on the parasitic power consumption of a liquid hydrogen heavy-duty fuel cell truck. Simulation results showed that the total parasitic power consumption could be reduced by up to 15% under steady-state conditions. Yang et al. (2023) used the Chinese-World Transient Vehicle Cycle to simulate the annual energy savings of this system in heavy-duty fuel cell trucks in four representative cities in China. The research found that energy savings were possible even in extremely cold regions, with annual average energy savings ranging from 9 to 15% depending on the city. These findings demonstrate the potential of hydrogen and onboard cold energy utilization systems for reducing energy consumption and greenhouse gas emissions in heavy-duty transportation.

Researchers worldwide are developing new hydrogen technologies to address current issues and ensure economic and energy security. The United States Department of Energy has created a multi-year strategy with ambitious goals and benchmarks for developing hydrogen infrastructure, storage technologies, and fuel cells (Hamad et al. 2014; Lehmann et al. 2017). The cost of hydrogen is expected to range between 2 and 4 US dollars per kilogram, equivalent to one gallon of gasoline (Sazali 2020). Australia also increasingly favors hydrogen manufacturing, especially exportation (Boretti 2020). By splitting water molecules and coal gasification, hydrogen could be implemented in production using excess wind and solar energy. However, there are numerous obstacles to overcome in hydrogen production. Considerations must be made when converting syngas to hydrogen from natural gas or coal feedstock, especially regarding energy costs.

The microwave plasma source is a successful technology for producing hydrogen and was employed by NASA as the primary fuel source for its rocket propellant (Chehade et al. 2020). Due to its potential as an energy-efficient alternative source, researchers are studying hydrogen’s potential in various industries. It has been chosen as one of the options for primary sources in the automotive industry, particularly for fuel cell electric vehicles with a low-polluting structure (Sazali 2020). The electric vehicle industry also has various energy systems, such as rechargeable metal batteries with low environmental impact and high energy density lithium-selenium (He et al. 2020).

The polymer electrolyte membrane fuel cell is the most practical method for generating electricity through an electrochemical reaction in fuel cell electric vehicles. Besides, internal combustion engines powered by hydrogen are also common in the automotive industry, along with fuel cell electric vehicles. Hydrogen can be stored in gaseous or liquid form for use in automobiles (Sazali 2020). Many vehicles, such as Ford Zetec 2.0 L, Toyota Mirai fuel cell electric vehicles, FM fuel cell electric vehicles, Electro Van, GM HydrogenGen3, BMW 750 hl, and BMW Mini Hydrogen, use gaseous or liquid hydrogen as their primary fuel. Hybrid electric vehicles are the next generation of vehicles powered by hydrogen. However, their systems are different because, in internal combustion engines, the pressure created by the combusted hydrogen propels the system in a circular motion. In contrast, the hydrogen-based engine in hybrid electric vehicles rotates the wheels and recharges the vehicle’s batteries.

On the other hand, fuel cell electric vehicles operate differently from internal combustion engines as they solely rely on hydrogen fuel cells to produce electricity for vehicle batteries (Sazali 2020). Furthermore, there are reported differences in the volatile organic compounds’ emissions between internal combustion engines, hybrid electric vehicles, and fuel cell electric vehicles, with the latter having the lowest emissions (Ugurlu and Oztuna 2020). In light of this, the German state of Schleswig–Holstein plans to electrify its entire railway network by 2025 by utilizing fuel cell technology to power both the trains and the grids (Lipman 2020; Pollet et al. 2019). Similarly, Leeds in the UK has revealed its intention to create a “hydrogen city” by replacing natural gas with hydrogen in its pipes (Sovacool et al. 2019).

Japan has announced a 40-billion-year plan to install hydrogen technologies for the Tokyo 2020 Olympics, including hydrogen transportation via pipelines to fuel cells that will power the buildings on an industrial scale (Nguyen et al. 2019).

While renewable technologies for hydrogen production have been developed, economic and technical limitations have resulted in the predominance of non-renewable sources, such as fossil fuels, for hydrogen production. Natural gas reforming is expected to increase hydrogen generation in future, despite its carbon emissions (Dunn 2002; Sazali 2020). Consequently, there is a need for carbon dioxide capture and sequestration systems to mitigate these emissions. Developing hydrogen separation technologies may allow for simultaneous carbon dioxide capture and high-purity hydrogen production.

Korberg et al. (2023) conducted a study on the feasibility of using hydrogen directly in all energy sectors through hourly energy system analysis for a 100% renewable energy system for Europe in 2050. The results indicated that using hydrogen for heating purposes is not cost-effective and inefficient. While hydrogen for electricity production can reduce biomass consumption, the high system losses result in increased costs. Hydrogen is more expensive than liquid e-fuels and electrified transportation in the transportation sector due to high infrastructure costs and low energy efficiency. However, hydrogen could benefit the industrial sector by decreasing biomass consumption at a lower cost than other energy sources. Still, electrification and e-methane may be more practical alternatives. From a systems perspective, hydrogen will be crucial for future renewable energy systems, primarily serving as an e-fuel feedstock rather than a direct end-fuel in industries that are challenging to regulate.

## Artificial intelligence for sustainable energy systems

**Fig. 5:**
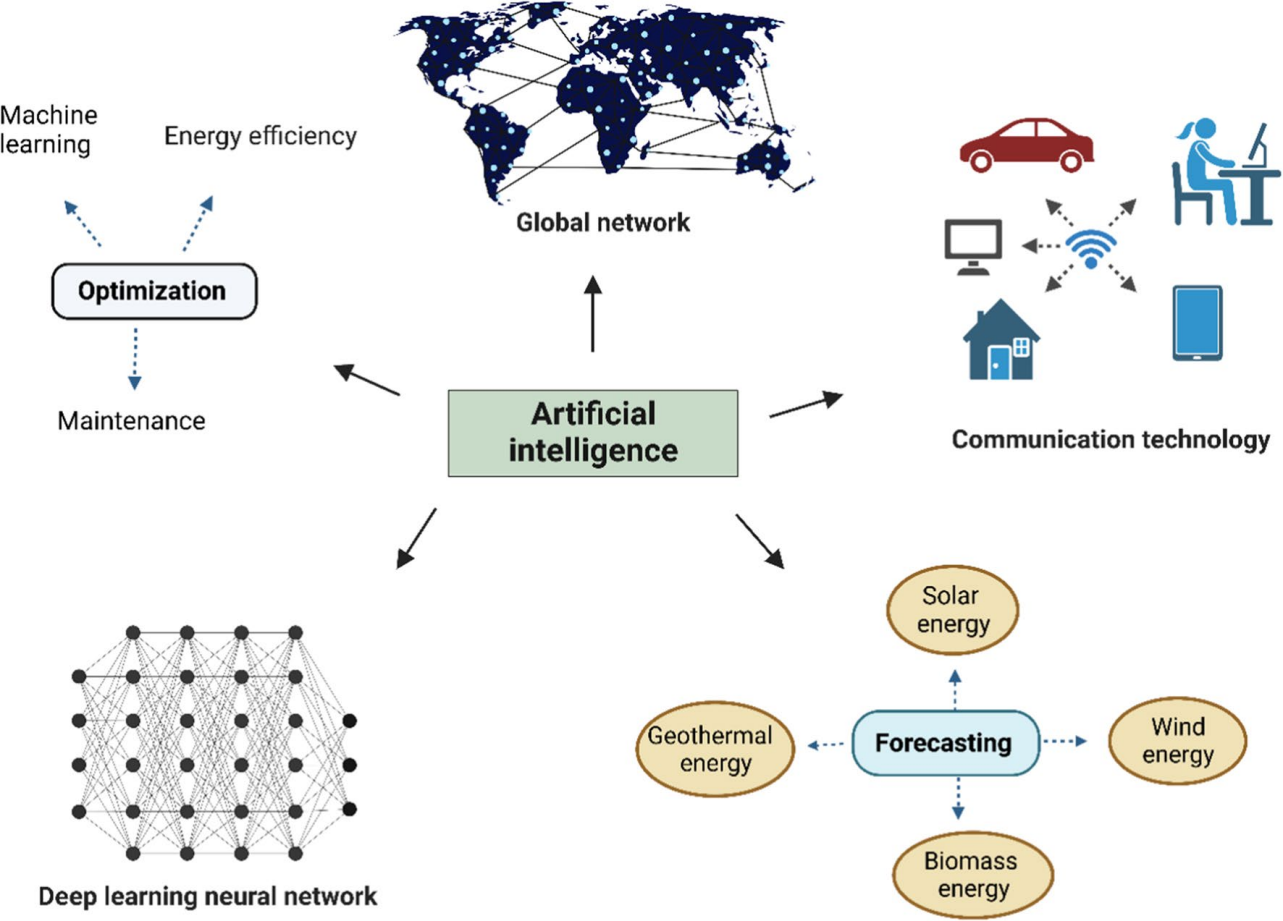
Role of artificial intelligence in energy saving. The potential for artificial intelligence to optimize energy efficiency, machine maintenance, and learning processes is vast. In addition, artificial intelligence can improve connectivity between applications to reduce energy consumption in homes, workplaces, and transportation systems. In order to maximize the use of renewable energy sources, artificial intelligence can also help with weather forecasting and other relevant parameters. By utilizing deep neural networking, artificial intelligence can function as a small village and effectively keep the world informed of the most recent information

The emergence of digital technologies has the potential to significantly transform the way we generate, trade, and consume energy. Artificial intelligence technology underpins the new digitalization model, which enables intelligent software to optimize decision-making and operations, automatically integrating energy supply, demand, and renewable sources into the power grid, as shown in Fig. 5. Supercomputers, power electronics, cyber technologies, and bi-directional connectivity between the control center and equipment are sophisticated infrastructures available to the smart energy sector, replacing old, ineffective, outdated, and unreliable traditional power systems that lack adequate protection from fault circumstances (Bose 2017). However, integrating renewable energy sources into the electricity system poses challenges due to their variable nature, which the traditional power system was not designed to manage. Recent advancements in artificial intelligence technologies, including machine learning, deep learning, IoT, and big data, are changing the energy sector (Fig. 5). Many countries have adopted artificial intelligence technology for managing, forecasting, and running efficient power systems (Kow et al. 2016; Yang et al. 2019). Artificial intelligence also enables efficient control of photovoltaic systems (Youssef et al. 2017).

Predictive technologies are commonly used to estimate the generation of both fossil fuels (such as oil, natural gas, and coal) and renewable energy sources (such as wind, hydro, solar, and geothermal energy), as well as load demand and electricity costs. These forecasts serve various purposes, including fuel purchases, generation planning, distribution schedules, investment programs, maintenance schedules, and security purposes. Artificial intelligence has played a significant role in load demand planning and forecasting (Kong et al. 2018), solar energy forecasting (Rodríguez et al. 2018), wind energy forecasting (Zhang et al. 2021), and hydro and geothermal energy forecasting (Lateef et al. 2022) (Fig. 5). Ahmad et al. (2021) have demonstrated how artificial intelligence techniques can outperform conventional models in computing efficiency, controllability, data handling, cyberattack prevention, optimization of energy efficiency and predictive maintenance control. The application of big data, machine learning, and artificial intelligence is expected to impact the energy market in future significantly. These findings highlight the critical role that artificial intelligence can play as an enabler of a sophisticated, data-driven energy industry, providing a powerful tool to enhance operational performance and competitiveness. To achieve tangible results and remain competitive, energy sector stakeholders, including utilities, power system operators, and independent power producers, may need to prioritize adopting artificial intelligence technologies.

Artificial intelligence has superseded the previous decision-making systems that relied on precedent and regulations. By optimizing goals and maximizing interests, artificial intelligence is better suited to meet the demands of modern civilization, such as energy conservation, environmental protection, and efficient engineering construction (He et al. 2022).

Table 3 presents different areas in which artificial intelligence can be utilized to save energy. In this context, Chen et al. (2022a) implemented artificial intelligence to conserve energy in these areas. The researchers analyzed 164 research articles and 113 artificial intelligence techniques to develop a standardized methodology for identifying practical methods for energy conservation through machine learning. The universal workflow resulted in energy savings of 35% in building energy costs, 25% in heating, air conditioning, and ventilation equipment systems, a 50% reduction in artificial lighting systems, up to a 70% reduction in information transfer and communication power, a continuous output of 30% peak power from renewable energy devices to the microgrid, and a 20% reduction in factory power demand after careful analysis of experimental data. This study’s universal workflow provides a practical approach for using artificial intelligence in various applications, considering factors such as application technology selection, qualitative and quantitative benefits, and control response variations. Practical design recommendations can speed up the adoption of artificial intelligence for commercial energy conservation purposes.

**Table 3:** Uses of artificial intelligence technology in energy saving

Data type	Artificial intelligence technology			Effect		Reference
	Learning	Optimization	Control	Energy saving	Other benefits	
Time series and text data	Building energy analysis model, multi-layer perception, multi-layer perception	Not reported	Model-based predictive control multi-layer perception	42.6% in building energy management systems	Model-based predictive control up to 6.3%	Salakij et al. (2016)
Time series data	Bayesian network, support vector machine, reward	Not reported	Scheduling model-based predictive control	35% in building energy management systems	Not reported	Lima et al. (2015)
Text data	Simulation model, multi-layer perception	Multi-objective genetic algorithm and hybrid multi-objective genetic algorithm	Multi-agent control system	31.6% in building energy management systems	8.1% thermal comfort index improvement using a hybrid multi-objective genetic algorithm	Shaikh et al. (2016)
Time series data	Multi-layer perception	Genetic algorithm	Scheduling control	25% in building energy management systems	Not reported	Yuce and Rezgui (2017)
Time series data	Recurrent neural network	Not reported	Multi-agent control system	19.8% in building energy management systems	Not reported	Javed et al. (2018)
Time series data	Deep neural network	Not reported	Air handling unit controller (on/off)	18.97% in building energy management systems	Not reported	Jafarinejad et al. (2019)
Time series data	Distributed energy resource model, feedforward neural network	Mixed-integer linear programming	Home automation controller	Not reported	6.2% low energy cost	Hosseinnezhad et al. (2020)
Time series data	Q-learning network	Policy gradient value gradient	Control strategy	Not reported	15% cost saving in Heating, Ventilation, and Air Conditioning	Du et al. (2021)
Text data	Occupant feedback, classifier	Not reported	Model-free predictive control	60% in heating, ventilation, and air conditioning	Not reported	Purdon et al. (2013)
Time series and Text data	White box model, feedforward neural network	The mixed-integer model predictive controller	Model-based predictive control	50% saving in renewable energy sources	50% cost saving	Mayer et al. (2016)
Text data	Vehicle-to-grid model, feedforward neural network	Artificial bee colony, particle swarm optimization	Charging controller	10% saving in renewable energy systems incorporating plug-in-hybrid electric vehicles	System operational conditions improvement	El-Zonkoly (2014)
Text data	MATLAB model/ feedforward neural network	Not reported	Fuzzy and control	8.5% saving in the electro-hydraulic industrial system	Not reported	

Integrated building energy management systems, assisted control of heating, ventilation, and air conditioning systems, industrial energy conservation, information and communication technology, artificial lighting systems, power systems, electric power grids, renewable energy systems, and transportation are examples of energy saving applications of artificial intelligence

Aziz et al. (2013) conducted a study on smart meters, which logged human activity within households and the power consumption of each appliance. The study introduced an artificial intelligence-based meter that categorizes appliances into three groups based on their energy consumption levels. The meter then provides customers with various energy quotas for each group, allowing them to manage their energy usage more effectively. In addition, the meter sends alerts to homeowners via mobile phone or email when energy consumption approaches 90% of the selected quota, thus improving the dependability and power quality of the intelligent meter.

Lee and Cheng (2016) conducted a study that reviewed 305 energy management systems that utilized artificial intelligence for industrial energy savings and equipment energy savings in building energy management systems. According to their findings, the building’s lighting systems can contribute up to 39.5% of its energy savings when using an expert system. The energy savings for buildings and industry ranged from 11.39 to 16.22 and 10.35 to 18.89%, respectively. Additionally, between 14.07 and 16.66% of energy was saved via heating, ventilation, air conditioning, and other equipment. Cotrufo et al. (2020) studied the development and application of predictive model control in institutional buildings. The model predictive control methodology utilizes artificial intelligence to construct models. It can lower building heating demand by 4.3% and reduce natural gas consumption and greenhouse gas emissions by approximately 22% compared to traditional control methods. Similarly, Lee and Tsai (2020) found that the entire building can experience energy savings of 13.7–18% when comparing model-based predictive control to conventional control systems.

Various mathematical algorithms, including heuristic algorithms such as the shuffled frog-leaping algorithm (SFLA), firefly optimization algorithm (FFA), gravitational search algorithm (GSA), artificial immune system (AIS), particle swarm optimization, ant bee colony optimization, teaching–learning-based optimization, ant colony optimization (ACO), cuckoo search (CS), bacterial foraging optimization (BFO), and coral reef optimization, can be used as intelligent optimization techniques for energy conservation. (Cui et al. 2017; Mamun et al. 2020). The studies have shown that multi-objective optimization using these techniques can decrease energy usage, carbon dioxide emissions, and operational costs.

**Fig. 6:**
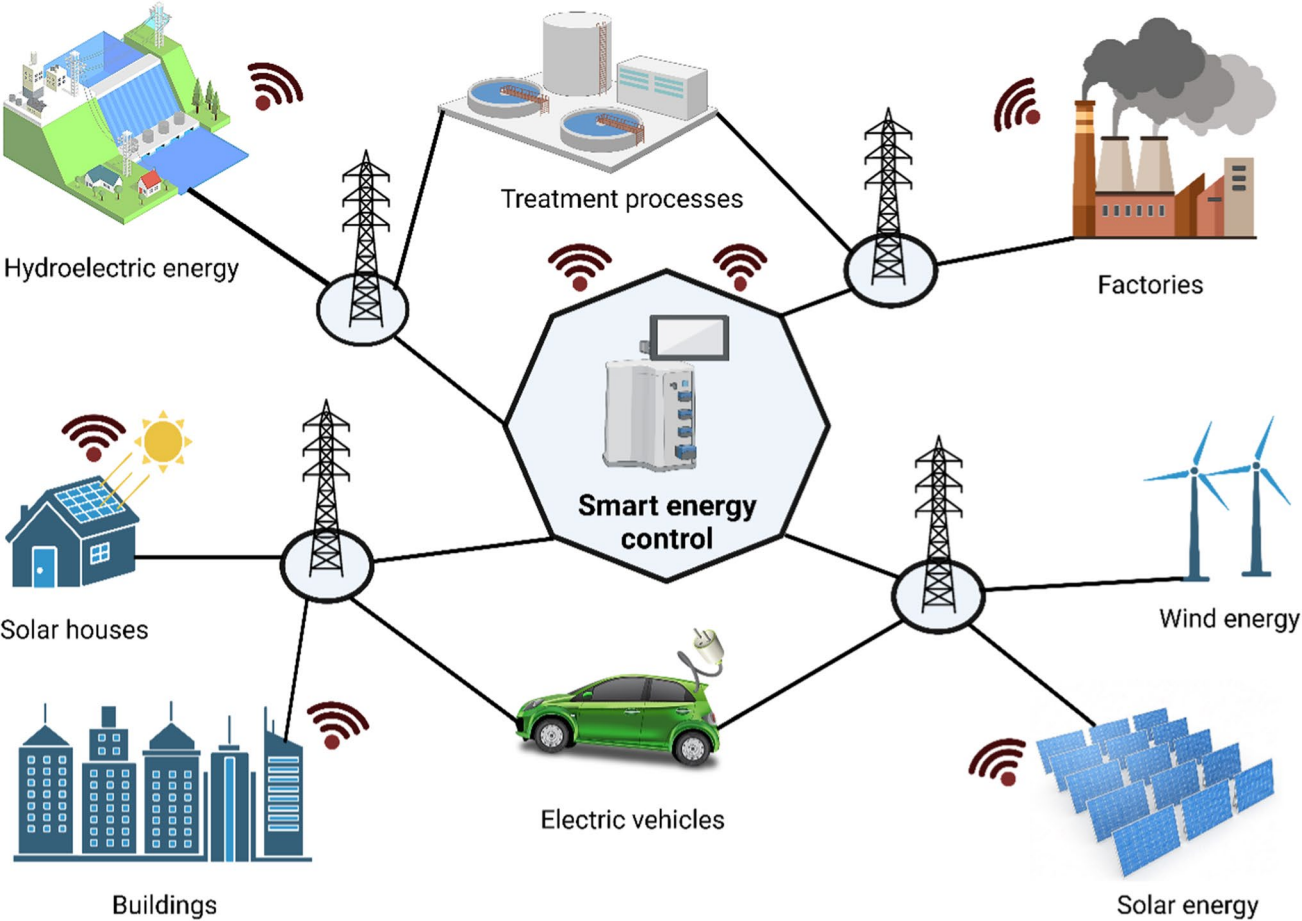
Potential applications of artificial intelligence in the electric sector. In the electricity industry, artificial intelligence offers numerous possibilities. It has the potential to automatically optimize power generation, distribution, and transmission operations, balance the grid without human intervention, make quick trading and arbitrage decisions, and eliminate the need for manual temperature adjustments or supplier searches. These technologies can enhance the ability of electric utilities to predict supply and demand, save energy, balance the grid in real-time, reduce downtime, maximize yield, and improve the end-user experience

Figure 6. illustrates the use of artificial intelligence for renewable energy and its potential future applications. The electric utilities industry is well-positioned to adopt artificial intelligence shortly. At every stage of the value chain, including power generation and end consumers, there are opportunities for machine learning, robotics, and decision-making automation. These technologies could assist electric utilities in forecasting supply and demand, balancing the grid in real-time, minimizing downtime, maximizing yield, and enhancing end-user experience. Jha et al. (2017) reviewed 372 papers to analyze various energy sources: wind, solar, geothermal, hydroelectric, ocean, biological, hydrogen, and hybrid. The results showed that hybrid artificial intelligence systems were more effective than single artificial intelligence techniques for analyzing renewable energy sources. In another study, Thieblemont et al. (2017) analyzed multiple model predictive control studies to increase energy savings through predictive control in weather forecasting. The energy cost savings ranged from 1.5 to 47%, and the benefits of cost savings ranged from 0 to 52%.

Building an energy conservation framework utilizing the artificial intelligence technique was implemented by Lee et al. (2020). Their approach involved a three-level building energy savings strategy that integrated five-category artificial intelligence control tools. The three levels consisted of equipment-level control, facility-level control, and entire building energy savings. It was found that motor power and air conditioning systems contributed to 70% of energy usage, lighting systems to 20%, and plug the power and office automation equipment to the remaining 10%. Before implementing the artificial intelligence framework, the annual electricity cost was US$1,004,339. After implementing the framework, energy consumption was reduced by 47.5%, 37%, and 36.9% at the equipment, facility, and overall building levels, respectively, resulting in savings of up to US$385,203. Similarly, Sha et al. (2019) demonstrated that utilizing artificial intelligence-assisted optimization based on building information models led to a 22.7–25% decrease in heating, ventilation, and air conditioning energy consumption.

City-wide artificial intelligence applications were examined, revealing potential energy savings of around 30% through smart energy management, using embedded computational intelligence algorithms in sensors such as distributed artificial intelligence and edge computing. Solar power installation costs have been reduced by 50%, and using artificial intelligence optimization has resulted in economic gains of 11–9%, with an expected reduction in carbon emissions by 11%. Additionally, machine learning was utilized to analyze data from multiple social media and traffic reports, resulting in a 10% reduction in average travel time (O’Dwyer et al. 2019).

In the oil and gas upstream industry, Koroteev and Tekic (2021) explored the application of artificial intelligence in tasks such as geological evaluation, drilling, reservoir engineering, and production optimization. Combining real-time drilling telemetry with machine learning resulted in savings of up to 20% in time and 15% in costs for detecting drilled rock types and potential failures. The best approach led to a growth in the marginality of up to 20% on campaign investment.

## Individual role in energy saving

Energy savings at the individual or household level can contribute significantly to decreases in energy demand and associated emissions, provided that policies are implemented to encourage and support people in changing their behavior. A 10-point plan (playing my part deal) to reduce the European Union’s dependence on Russian natural gas and cut oil use in March and April 2022 was published by the International Energy Agency in response to Russia’s invasion of Ukraine. The plan’s full implementation would result in annual oil and natural gas savings of 220 million barrels and approximately 17 billion cubic meters, respectively, lowering annual energy costs for households by more than €450 on average (IEA 2022b).

**Fig. 7:**
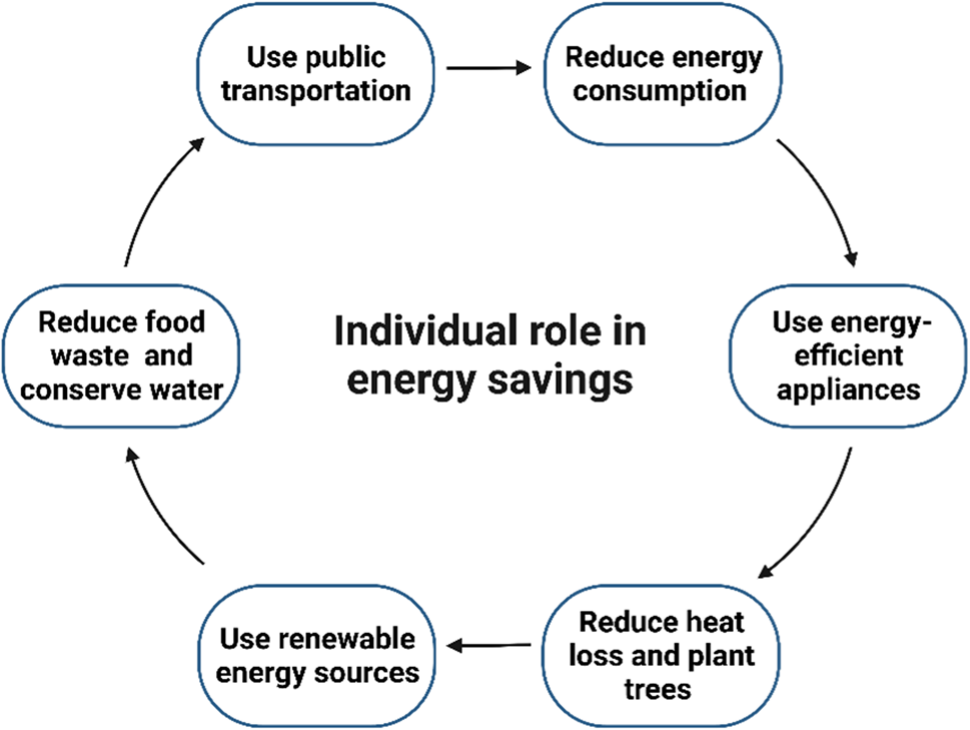
Role of individuals in energy saving. This plan outlines individuals' actions to save energy, reduce carbon footprint, and improve the environment. It highlights the importance of energy efficiency measures such as using energy-efficient light bulbs and appliances, upgrading insulation and windows, and supporting energy-efficient policies and programs. In addition, the plan promotes sustainable transportation options such as walking, bicycling, carpooling, and the use of renewable energy sources such as solar and wind. Additionally, the plan recommends water conservation, reducing food waste, planting trees and vegetation to provide shade, promoting energy conservation, and reducing reliance on Russian natural gas

Citizens can significantly contribute to achieving these reductions—most of the suggestions in playing my part deal with altering behavior. In the past, particularly during the 1970s oil crises and as part of Japan’s response to the Fukushima disaster, behavioral change has been sought as part of crisis response with success. The European Union’s ten member states have already implemented policies to support the behavioral adjustments outlined in the playing my part deal. For instance, the Netherlands and Italy are encouraging households to lower their thermostats while at the same time requiring temperatures to be moderated in public, Germany and Austria have lowered the cost of public transportation, Belgium is encouraging car sharing, and so on (IEA 2022b). Behavioral changes could immediately save 0.6 million barrels per day of oil, 17 billion cubic meters of gas, and 30 TWh of electricity a year (IEA 2022b). The playing my part deal is illustrated in Fig. 7.

## Case studies of energy savings 

### The German 100% renewable energy switch by 2050

Climate policies have been implemented recently to respond to rising global temperatures and reduce carbon dioxide emissions. These policies include the German energy transition (Energiewende) in the early 2010s and the Paris agreement in 2015 (21st Conference of the Parties of the UNFCCC, 2015) (Hansen et al. 2019). However, achieving these goals is challenging, as it involves considering factors such as energy resource availability and the costs of international cooperation. The Energiewende, initiated in 2010, resulted from extensive energy policy discussions in Germany, driven by various events and factors, including the need to phase out nuclear power and ambitious climate targets (Hake et al. 2015). The lengthy transition has highlighted the importance of long-term planning in the energy sector with its complex infrastructure (Renn and Marshall 2016). The Energiewende goals include increasing the share of renewable energy in final energy to 60% and electricity demands to 80% by 2050 while reducing greenhouse gas emissions by 80–95%.

Targets have also been set for decreasing primary energy demand and electricity, heat, and transportation needs (Pregger et al. 2013). However, previous strategies have focused more on creating an Energiewende policy than designing the energy infrastructure to meet the emission targets. This underscores the crucial role of policies in shaping future energy development. Additionally, the current legal framework does not facilitate the transition to Energiewende aims, particularly in the heating market (Bauermann 2016).

Many studies have focused on various aspects of Germany’s Energiewende. Some studies have examined its regional implementation (Lutz et al. 2017), while others have explored future wind power sales designs (Anatolitis and Welisch 2017). Schmid et al. (2016) have emphasized the importance of involving institutions and actors in defining the future energy system, especially when introducing new technologies. Specific areas of the energy system, such as transportation and electric cars (Canzler and Wittowsky 2016; Schill and Gerbaulet 2015), the heating market (Bauermann 2016), and the future evolution of the German electricity market (Knaut et al. 2016) have also been the focus of several studies.

To implement the Energiewende in Germany, several studies have focused on the electrical sector while excluding other energy sectors. For instance, Schmid et al. (2013) analyzed five studies solely on the electrical sector. Similarly, Lehmann and Nowakowski (2014) examined three scenarios for constructing the future renewable electricity industry, emphasizing the electrical sector. These scenarios included a decentralized, centralized, and pan-European strategy for incorporating more renewable energy sources. Additionally, Gullberg et al. (2014) assessed the benefits of closer linkages between the Norwegian and German energy systems to utilize Norwegian hydropower capacity as electricity storage reserves. Other studies have identified technical obstacles qualitatively by highlighting bottlenecks that must be addressed before a successful energy transition is possible (Scholz et al. 2014). To address this gap, Schroeder et al. (2013) proposed a comprehensive energy transition scenario that covers all sectors of the energy system, including electricity, heat, and transportation. Their scenario includes using various renewable energy sources and energy efficiency measures to achieve the Energiewende targets. Other studies developed a model-based approach to assess the feasibility of the Energiewende targets and identify the necessary technological and policy measures for achieving them. They concluded that a comprehensive energy system transformation is required, including electrifying the heating and transportation sectors and expanding the electricity grid. These studies highlight the importance of taking a holistic, multi-sectoral approach to the Energiewende to identify effective solutions for achieving the ambitious climate and energy targets (Henning and Palzer 2014; Pregger et al. 2013).

Henning and Palzer (2014) proposed a transition to 100% renewable energy in the heating and electricity sectors while keeping energy system costs comparable to current prices. They demonstrated that this transition is achievable, even with limitations on biomass and renewable electricity sources. Similarly, Pregger et al. (2013) highlighted the importance of reducing fuel imports and costs to lower overall energy system expenses. Both studies underscore the need to decrease energy demands to meet climate targets, focusing on the heating sector, which presents significant opportunities for energy savings (e.g., reducing heat demand by 60%).

Various studies present plans for achieving 100% renewable energy nationally across all sectors, going beyond the solutions proposed for Germany. Examples can be found in countries such as the European Union (Connolly et al. 2016; Krajačić et al. 2011), Sweden (Bramstoft and Skytte 2018), Macedonia (Ćosić et al. 2012), Finland (Child and Breyer 2016), and the UK (Hooker-Stroud et al. 2014). Other countries such as Brazil (Gils et al. 2017), Australia (Blakers et al. 2017), Portugal (Nunes et al. 2015), New Zealand (Mason et al. 2010), and Turkey (Kilickaplan et al. 2017) provide examples of high-renewable studies. Still, they do not cover the entire energy system. However, as previously mentioned, no complete energy system evaluations for Germany have been conducted. Previous studies have focused on limited sectors, specialized technology, and investment optimization strategies. In contrast, Hansen et al. (2019) proposed a comprehensive strategy for achieving 100% renewable energy in the German energy system. Their approach evaluates the most crucial measures for achieving these targets while considering resource potentials and costs. The strategy aims to achieve a net 100% renewable energy share over a year by 2050, exceeding the Energiewende targets in terms of renewable energy share. This study improves and expands upon the methods developed by Connolly et al. (2016) by examining the significance of multiple measures, including an in-depth analysis of the transportation sector and their implementation in the energy system.

Energy efficiency is essential for preserving the sustainability of biomass and renewable electrical resources. The heating sector has significant energy saving potential; additional savings are also possible in the industrial and electrical sectors. Moreover, various technologies, such as heat pumps, electrolyzers, and electric vehicles, increase the energy system’s flexibility and efficiency and facilitate the integration of more renewable electricity sources. Given the widespread electrification of all energy sectors, all available renewable electricity resources should be installed. However, the biggest challenges in achieving this transition are related to the limited availability of biomass resources. Even when all variable electricity potentials are installed, the 100% renewable energy scenario with the lowest biomass demand depends on hydrogen for thermal plants' transportation, gasification, and electro-fuel production. Nevertheless, this scenario is only slightly above the specified biomass threshold of 400 TWh/year, and the primary obstacle is the limited availability of biomass resources. Alternative scenarios that involve meeting all transportation needs with electricity, utilizing second-generation biofuels, bio-electro-fuels, or carbon dioxide electro-fuels require higher biomass consumption due to increased electricity needs or fuel production.

Additionally, limitations on excess electricity production lead to a significant rise in biomass demand. Balancing between high biomass consumption and excess electricity production from variable sources is a necessary compromise. However, some of these scenarios are speculative and require significant technological advancements and infrastructure modifications to be feasible in a future energy system. As a result, constructing a 100% renewable energy system in Germany still poses significant challenges.

### Compressed air energy storage in China

Managing energy generation and load demand, adjusting to peak load demand, and providing backup power in case of power supply breakdowns are all critical roles of energy storage in power systems (Venkataramani et al. 2016). Compressed air energy storage has emerged as one of the most efficient techniques for large-scale energy storage, alongside pumped hydro energy storage (Argyrou et al. 2018). Compressed air energy storage technology was initially developed as an alternative to pumped hydro energy storage, primarily for storing off-peak electricity and transferring it to peak load hours. In addition to this, compressed air energy storage offers a range of ancillary services such as frequency control, reserve, and reactive power regulation for grid systems, thanks to its quick start-up, broad working conditions, and reactive support capabilities (Lund and Salgi 2009; Tong et al. 2021). Therefore, this section focuses on compressed air energy storage as an importance energy storage technology.

#### Research focus

Since 2010, China has surpassed the USA to become the world’s largest energy consumer, as reported by the International Energy Agency (Tong et al. 2021). The country’s increasing focus on environmental issues such as air pollution and the greenhouse effect has resulted in renewable energy emerging as China’s fastest-growing energy source. As of 2017, wind and solar renewable energy accounted for 16% of China’s installed capacity and contributed 7% of the country’s overall power generation (British-Petroleum-Company. 2018). The country’s renewable energy supply is expected to rise to nearly 20% by 2030, highlighting its significant potential for growth in renewable power generation.

Although China has made significant strides in renewable energy generation, the commercialization of energy storage is still in its early stages. As of 2017, China’s cumulative installed energy storage capacity was 28.9 gigawatts (GW), which is only 1.6% of the country’s total power-generating capacity of 1777 gigawatts (Tong et al. 2021). This falls short of the state grid’s target of 4–5% by 2020 (Li et al. 2015). Pumped hydro energy storage comprises 99% of China’s total installed energy storage capacity, with electrochemical energy storage rapidly developing in recent years. However, most electrochemical energy storage projects and pumped hydro energy storage stations are concentrated in central and eastern China (Tong et al. 2021). The “Three North” regions of China, where most of the renewable energy is used, face geographical constraints that limit the development of pumped hydro energy storage (Zhang et al. 2017a; Ming et al. 2013).

Using compressed air for energy storage has been around since the 1940s, and the technology is based on gas turbine principles (Tong et al. 2021). The traditional compressed air energy storage system operates on a simple principle. During the charging process, the compressor converts excess electrical energy into the internal energy of high-pressure air for storage. During discharge, the high-pressure air is released to power the turbine generator, transforming the internal energy of the compressed air back into electrical energy (Budt et al. 2016). This method is known as adiabatic compressed air energy storage. The compression heat is dissipated through air cooling, and fuel combustion is necessary to heat the compressed air at the expander’s input. The cycle efficiency of adiabatic compressed air energy storage is approximately 50% (Rogers et al. 2014). This method involves storing electrical energy in high-pressure air, which can then be expanded to generate electricity as needed.

Compressed air energy storage offers several advantages, including high reliability, cost-effectiveness, flexibility in layout, and minimal environmental impact (Venkataramani et al. 2016). Furthermore, combining compressed air energy storage with renewable energy sources can help to address the challenges associated with renewable energy integration (Li et al. 2018).

Chinese academics have extensively studied the technology and applications of compressed air energy storage in recent years. They have introduced a new type of compressed air energy storage system called supercritical compressed air energy storage (Guo et al. 2017; Liu et al. 2014a; Mei et al. 2015; Zhang et al. 2017c; Zhao et al. 2015a). In addition, they have conducted theoretical and experimental research on non-supplementary fired compressed air energy storage technology (Mei et al. 2017; Xue et al. 2016a; Xue et al. 2016b).

Compressed air energy storage is one of the most suitable methods for grid-connected storage. It can be profitable for load-shifting applications based on the price differential between peak and valley electricity. Furthermore, compressed air energy storage is an attractive option due to the rapid growth of renewable energy, such as wind and solar energy. It can be used to mitigate the adverse effects of intermittent and fluctuating renewable power output on the power system (Tong et al. 2021). Extensive research has been conducted on integrating large-scale wind power and compressed air energy storage to improve the quality and controllability of wind power. Various integrated solutions have been studied, such as centralized/distributed, series/parallel, and different energy discharge modes (Madlener and Latz 2013; Hasan et al. 2016; Zhao et al. 2016). Thus, compressed air energy storage can provide a practical solution for integrating renewable energy (Facci et al. 2015; Tong et al. 2020).

Moreover, compressed air energy storage has attracted significant attention in distributed energy supply systems due to its ability to simultaneously generate electricity, heat, and cold (Facci et al. 2015; Mohammadi et al. 2017). To address the low electric conversion efficiency of compressed air energy storage, poly-generation can achieve high overall energy usage efficiency, with potential applications in transitioning from centralized distribution to diverse, dispersed, and intelligent development (Tong et al. 2021). Researchers have also focused on using compressed air energy storage in trigeneration and hybrid energy storage systems that combine compressed air energy storage and flywheel energy storage (Yao et al. 2016; Yao et al. 2017; Zhao et al. 2014; Zhao et al. 2016; Zhao et al. 2015b).

Han and colleagues' research primarily focused on the operation control of an advanced adiabatic compressed air energy storage system and its application in trigeneration systems (Han and Guo 2018a, b; He et al. 2019). In contrast, Yan and colleagues investigated using compressed air energy storage for trigeneration and operation control of composite energy storage systems in microgrids (Yan et al. 2017; Yan et al. 2018). Furthermore, the authors investigated the integration of adiabatic compressed air energy storage with wind and solar energy (Chen et al. 2017; Fan et al. 2018).

#### Applications of compressed air energy storage

This section covers the applications of compressed air energy storage in three areas: grid control, renewable energy generation, and demand-side management.

##### Grid control

In recent years, China’s power system has experienced an increase in the peak-to-valley difference, leading to lower utilization of electricity generation equipment (Li et al. 2019). Additionally, the growing use of intermittent and fluctuating renewable power generation has resulted in higher demands for grid regulation services to ensure reliable operation. However, due to the dominance of coal-fired power generation in China’s power production, there is a significant lack of grid regulatory capability. To address this issue, centralized large-scale compressed air energy storage systems with several hundred megawatts unit capacities and several hours of production capacities can provide grid regulation services.

Using large-scale compressed air energy storage in grid regulation can help alleviate the lack of grid regulation capabilities and reduce the partial operation of coal-fired power plants, resulting in increased power generation efficiency. To this end, Chang et al. (2015) conducted a techno-economic analysis of compressed air energy storage peak-shaving power plants using the peak-to-valley electricity price mechanism. Their research highlights the importance of a reasonable subsidy policy in reducing economic risk and improving the return level of compressed air energy storage power plants. Li et al. (2019) examined the reserve capacity model of compressed air energy storage systems and their role in providing reserve services. They found that grid regulation using compressed air storage can lower system energy and reserve costs. However, they suggest that compressed air energy storage may better suit day-ahead scheduling rather than real-time dispatch. Additionally, Liu et al. (2014b) proposed using coal-fired compressed air energy storage to address China’s energy needs, given the country’s abundance of coal but a scarcity of natural gas. Their techno-economic analysis suggests that using coal as a fuel rather than natural gas can increase the economic viability of conventional compressed air energy storage.

##### Renewable power generation

Integrating energy storage into renewable energy can enhance output regulation capability and improve power quality. This is an effective method to increase renewable energy utilization in power generation and alleviate China’s challenges in accommodating renewable energy sources. Compressed air energy storage integration with renewable power generation primarily focuses on large-scale centralized wind farms. Yang et al. (2014) proposed using electrical heaters to increase the thermal energy storage temperature by absorbing more wind power, thereby improving the economic viability of advanced adiabatic compressed air energy storage combined with wind power. The modified system was demonstrated to achieve wind farm regulation on a smaller scale than traditional advanced adiabatic compressed air energy storage.

Chen et al. (2017) explored the potential of a hybrid wind-solar-compressed air energy storage system to maximize the utilization of renewable energy in regions with abundant wind and solar resources. The hybrid system modifies the mature adiabatic compressed air energy storage technology, which replaces fuel combustion with a solar collector as a heat source for the energy release subsystem, utilizing large-scale wind energy as an input source. This enables the fluctuating wind energy to be transformed into high-quality, manageable electric energy, significantly increasing the share of wind energy in the power grid system beyond the conventional limit of 40% to over 80%. Coastal and offshore renewable energy sources, such as offshore wind, solar, current, and wave energy, are expected to play a crucial role in China’s future power generation (Kaminski and Rigo 2020; Wang et al. 2017b). Underwater compressed air energy storage has been identified as a promising solution to support the development of intermittent coastal and offshore renewable energy sources, providing an efficient and cost-effective energy storage unit. Wang et al. (2017b) compared underwater and underground compressed air energy storage systems for integrating offshore wind farms and found that underwater compressed air energy storage has the advantage of higher efficiency and energy storage density.

##### Demand-side management 

China’s power system has experienced a recent increase in capacity to meet the growing electricity demand. However, there has been a continuous decline in the average hours of equipment used for power generation, indicating an oversupply in the electrical market. This trend is attributed mainly to the substantial rise in installed renewable energy generation capacity. Demand-side management and energy storage have become key components of China’s power system reform to address this issue, focusing on diversifying supply and demand (Zhang et al. 2017b). Compared to other energy storage technologies like batteries, compressed air energy storage stands out due to its trigeneration capability (Li et al. 2012). The adiabatic compressed air energy storage system can generate combined heating, cooling, and electricity by utilizing excess electricity from the power grid. The expansion of compressed air can directly produce cooling power. At the same time, the recovery of compression heat can generate heating power, making it suitable for use in factories or public buildings in urban areas. Lv et al. (2017) proposed a novel trigeneration system based on adiabatic compressed air energy storage technology for shifting electrical energy peak loads. A case study showed that this system is more cost-effective than traditional compressed air energy storage systems and can achieve efficient and flexible energy use.

In recent years, using compressed air energy storage in combination with gas turbines based on combined cooling, heating, and power systems has been extensively studied. Meanwhile, Yang et al. (2017) and Wang et al. (2018b) studied the utilization of compressed air energy storage in combination with a gas turbine for combined cooling, heating, and power systems. During off-peak hours, excess power generated by the system is stored in compressed air energy storage, which is discharged during peak load times. Solar energy is used to heat high-pressure air during discharge, boosting the storage’s output power. Compressed air energy storage effectively addresses the efficiency decline during part-load and ensures reliable microgrid operation in distributed power supply with renewables. Li and Dai (2015) developed a small-scale compressed air energy storage system that is powered by a wind turbine, which provides stable electricity to microgrids. However, compressed air energy storage has a minute-level response time, and therefore, alternative energy storage technologies with fast response capabilities are necessary for real-time response requirements. To this end, hybrid energy storage systems that combine compressed air energy storage with supercapacitors, flywheel energy storage, and other storage technologies have been proposed by various researchers, including Huang and Ruixiang (2015) and Zhao et al. (2014). These systems provide effective power control for wind power generation and reduce grid fluctuations while maximizing solar energy usage.

### Challenges

Despite China’s advantageous position and significant technological demand, the commercialization of compressed air energy storage has been hindered by both technical and economic factors.

#### Technical factors

Although existing compressed air energy storage demonstration projects have reached a cycle efficiency of about 60%, a significant gap exists between the current and ideal efficiency levels of over 90%. Compared to battery energy storage, which is more widely used in China’s commercial electrical energy storage sector, compressed air energy storage has lower system efficiency. Therefore, further research is needed to improve the system’s energy transfer and conversion performance, optimize thermodynamic system processes, develop high-performance components, and study optimal control strategies.

Despite the abundance of low-cost underground salt cavern resources for large-scale energy storage in China, their geographic limitations pose a challenge, particularly in the “Three North” regions, which have promising renewable energy alternatives. There have been few studies on large-scale, high-pressure air storage in mine tunnels and cavities, and there has been no commercial application yet. Therefore, additional research is needed to determine the technological and financial feasibility of this type of air storage to encourage its commercial deployment.

In areas without access to mine tunnels or underground salt cavern resources, the high cost of artificial air storage using pressure vessels presents a significant obstacle in developing commercial compressed air energy storage systems. Therefore, artificial air storage devices that can be flexibly organized, such as liquefied air storage, can be an effective and economically viable alternative for large-scale compressed air energy storage.

#### Economic factors 

Although compressed air energy storage offers several advantages for power systems and is a feasible economic option, its value has not been fully recognized in the current Chinese power market. Currently, the market only offers limited opportunities for determining the return on investment through the use of the Chinese government’s benefit evaluation approach for energy storage devices (Yu et al. 2017). Consequently, compressed air energy storage is considered an expensive option. Its value must be evaluated using an advanced benefit evaluation system considering economic, environmental, and safety benefits.

Furthermore, the value of compressed air energy storage is somewhat restricted when it is exclusively used for load balancing and peak shaving. Additional actions are required to ensure the flexibility of China’s power system as it continues to develop. This could include opening ancillary markets and expanding the grid, which could help lower the demand for energy storage (Facci et al. 2015). Commercializing compressed air energy storage in China depends on further power reform.

However, the lack of incentives for energy storage applications hinders the development of compressed air energy storage in China. Therefore, an appropriate subsidy program is necessary to boost capital investment and the economy. Implementing suitable energy storage subsidy policies early in the technology’s development could encourage commercializing compressed air energy storage.

## Implications for the environment and society

Reducing greenhouse gas emissions is a critical strategy in the global fight against climate change, and decarbonizing energy systems play a vital role in achieving this goal. In particular, implementing low-carbon heating systems can significantly reduce emissions associated with housing, especially in hard-to-insulate dwellings. Table 4 demonstrates that various low-carbon technologies, such as heat pumps, photovoltaic and biomass boilers, solar heating, solar to electric boilers, and compressed natural gas, have a significant impact on reducing emissions. By adopting these technologies, we can make substantial progress in reducing the carbon footprint of our energy systems and, ultimately, contribute to mitigating the effects of global warming.

**Table 4:** Impacts of energy efficiency technologies on the environment and society in various regions

Region	Buildings	Energy saving technology	Environmental impact	Social impact	Reference
USA	Texas State University	Replacement of T8 bulbs and high-pressure sodium lamps with light-emitting diodes (LEDs) Solar panel installation Motor replacement Variable frequency drive installation Pump replacement	Carbon reduction: 12,561.81 tons	Improved photometric performance and user comfort, coupled with reduced likelihood of mechanical breakdowns due to the installation of variable frequency drives	Mohammadalizadehkorde and Weaver (2020)
Italy	The residential area for 1000 residents	Geothermal heat pump system	Carbon reduction; decrease in acid potential: 34%; 16.7%	–	Bartolozzi et al. (2017)
		Biomass wood chip stove	46%, 8.3%	–	
		Natural gas boiler-adsorption chiller	11.3%, 33.4%	–	
Southeastern Romania	Multi-family housing in single-room apartments	Solar air heating system–natural gas boiler system	Carbon reduction: 44 tons/year (maximum of 44.25%)	–	Paraschiv et al. (2020)
Benevento, Italy	Residential buildings and restaurants	The electricity grid provides buildings	Carbon emissions: 102.8 tons/year	Direct installation of photovoltaic power plants on the roof can provide shared energy services to customers	Ceglia et al. (2022)
		Photovoltaic arrays are delivered to buildings through external producers	47.2 tons/year	–	
		Building direct installation of photovoltaic power plants	63.3 tons/year	–	
Southern Finland	Nearly zero-emission building residential building	Solar collectors, geothermal heat pumps, photovoltaic panels, and grid-connected	Carbon emissions: 2626 kg/year (50.8% reduction)	–	Vares et al. (2019)
		Solar collectors, geothermal heat pumps, photovoltaic panels, off-grid	4790 kg/year (10.3% reduction)	–	
		Solar collector	2912 kg/year (45.5% reduction)	–	
Chambéry, France	Residence	Gas boiler	Carbon reduction: 0%	Provides thermal comfort	Fitó et al. (2021)
		Biomass boiler	Carbon reduction: 1.76 tons/year		
		Grid–electric boiler	Carbon reduction: 1.34 tons/year		
		Photovoltaic panels–electric boilers	Carbon reduction: 1.55 tons/year		
		Grid–air source heat pumps	Carbon reduction: 1.68 tons/year		
		Photovoltaic–air source heat pump	Carbon reduction: 1.75 tons/year		
		Grid–geothermal heat pumps	Carbon reduction: 1.76 tons/year		
		Photovoltaic–geothermal heat pump	Carbon reduction: 1.81 tons/year		
		Solar collectors	Carbon reduction: 1.61 tons/year		
Italy	Six residential buildings and three academic buildings	Solar collectors, photovoltaic/thermal panels	Carbon emissions: 23.45 tons; 11.7% reduction	–	Rosato et al. (2019)
Barcelona	Sports Center	Solar flat plate–natural gas condensing boiler	Carbon emissions: 5047 kg/year	Not visible to people outside the housing, no visual impact, no risk of dropping to lower levels during installation and maintenance operations	Casanovas-Rubio and Armengou (2018)
		Solar flat panel-electric water heater	7,150 kg/year		
		Electric water heater	14,732 kg/year		
		Natural gas	10,399 kg/year		
Korea	Farm pigsty	Geothermal heat pumps and solar heating; artificial intelligence computing	Carbon reduction: 1501 kg; formaldehyde reduction: 0.01 ppm; particulate matter reduction: 2.09 µg/m^3^; helium concentration reduction: 0.09 ppm	Improve animal feeding comfort	Mun et al. (2022)
China	Three separate but continuous offices at Tsinghua University	Air source heat pump	–	Reduced risk of ventilation and fewer complaints of local discomfort in the shoulder	Lin et al. (2016)
		Radiators			
		Geothermal			

The table illustrates the benefits of various energy-efficient systems, including biomass boilers and stoves, hybrid heat pumps, geothermal heating, and solar photovoltaic systems with integrated electric boilers. These technologies effectively reduce carbon emissions and provide substantial social benefits by enhancing thermal comfort and minimizing energy maintenance risks. “–” is not mentioned

In their 2020 study, Mohammadalizadehkorde and Weaver (2020) evaluated 13 buildings on the Texas State University campus in the USA. They identified opportunities for reducing carbon emissions by replacing sustainable building energy equipment. Installing solar energy panels had the most significant positive impact on the environment, reducing carbon dioxide emissions by 2926.81 tons annually and improving photometric performance and users' well-being. Replacing the lighting system resulted in the most substantial reduction in carbon dioxide emissions, amounting to 5387.00 tons. To achieve the best energy efficiency, installing variable frequency drives can effectively regulate the operation of fans and pumps according to seasonal changes, thus minimizing the risk of mechanical failure (Viholainen et al. 2013).

A study by Bartolozzi et al. (2017) compared the effectiveness of geothermal heat pumps and biomass systems with natural gas boiler systems for centralized heating and conventional stand-alone electrical systems. The results showed that biomass wood chip stoves offered the most significant reduction in greenhouse gas emissions, with a 46% decrease. Ground source vertical closed-loop geothermal heat pump systems followed with a 34% reduction. On the other hand, the natural gas boiler-adsorption chiller system displayed the highest potential for reducing acid emissions, with a 33.4% reduction. These findings demonstrate the environmental sustainability potential of alternative renewable heating and cooling methods.

Paraschiv et al. (2020) suggested an enhancement to the original natural gas boiler system by incorporating a solar air heating system onto the walls of the building. This integration led to a maximum reduction of 44 tons/year in emissions during the operational lifespan, highlighting the effectiveness of renewable energy sources in decreasing energy consumption in buildings. Furthermore, the authors proposed the installation of photovoltaic plants on each building’s rooftop, allowing users to connect to a mini-grid (physical or virtual) and share energy services without any financial incentives. This approach also enhances social acceptability. Moreover, integrating the photovoltaic array through an external producer into the building’s energy strategy reduces 55.6 tons/year (54.1%) of emissions compared to electricity generated by linking to the grid, making it a more environmentally sustainable option.

According to Vares et al. (2019), using solar collectors and photovoltaic panels for on-site renewable energy production can significantly reduce greenhouse gas emissions when connected to the grid, with a reduction potential of 50.8%. This can be particularly beneficial for energy saving buildings. Fitó et al. (2021) compared the carbon reduction performance of ground source heat pumps and air source heat pumps. They found that ground source heat pumps perform better environmentally due to their ability to replace gas and grid-driven boilers. However, the environmental advantage of photovoltaic panels is insignificant in the absence of energy storage, making their performance almost equivalent to that of solar collectors.

Casanovas-Rubio and Armengou (2018) compared the environmental impact of different types of collectors for domestic hot water systems, including condensing gas boilers and electric water heaters. They found that serpentine flat plate collectors offer the most significant environmental benefits, resulting in a 5% reduction in greenhouse gas emissions. Additionally, these collectors have a smaller footprint and less space, providing users with a more comfortable experience without compromising visual aesthetics. The design solutions also ensure that the collectors are not installed on external walls or sloped roofs, reducing the risk of worker injury during installation and maintenance.

Geothermal heating can provide a more comfortable thermal environment for sedentary residents by directly utilizing sustainable geothermal energy to heat the foot regions to a greater degree, resulting in lower power consumption and greenhouse gas emissions (Zhao and You 2020). Lin et al. (2016) tested radiant heating systems in three offices and found that users were most satisfied with the geothermal system. This is due to its ability to provide a more balanced moderate airflow (19%) and minimize large temperature swings and steep temperature gradients, thus reducing the likelihood of drafts and localized complaints of discomfort from the foot.

In summary, energy-efficient system technologies can effectively support the reduction of carbon emissions by minimizing electrical and thermal energy waste during system operation. This helps to reduce the amount of carbon dioxide generated. Moreover, these technologies enhance visual and thermal comfort while reducing safety risks associated with system maintenance.

## Conclusion

The energy crisis significantly impacts society’s economic, political, and environmental factors. Russia supplies nearly half of the European Union’s gas imports and 40% of its total gas consumption in 2021, threatening energy security and affordability. To address this, member states plan to reduce gas demand sharply. Green alternatives like air and ground source heat pumps, biomass boilers, solar thermal panels, infrared heating panels, and geothermal heating can also make residential heating systems more sustainable and energy-efficient. Renewable energy sources offer the possibility of a solution to the crisis. To reduce energy demand, building codes and regulations should prioritize energy-efficient design and construction practices. Passive design strategies, including orientation, shading, natural ventilation, and high-performance building envelopes, can reduce the demand for active heating and cooling systems. Nevertheless, location, climate, the size of the house, and the availability of renewable energy sources influence the selection of the most suitable green heating solution.

We can achieve a more efficient and effective low-carbon energy system by integrating different sectors and optimizing their interactions. Energy-efficient appliances, smart building technologies, and renewable energy systems like solar and geothermal can reduce waste. Smart grid technologies like demand response and dynamic pricing can optimize energy use. This multi-sector approach creates sustainable growth opportunities. To achieve a sustainable and resilient future, we need a systemic approach recognizing that reducing energy demands and promoting renewables are only part of the solution.

The electrification of transportation systems, such as passenger vehicles and public transportation, can substantially reduce greenhouse gas emissions and improve air quality. Installation of charging infrastructure, such as public electric vehicle charging stations and battery-swapping stations, is crucial to facilitate this transition. Incorporating intelligent mobility technologies such as ride-sharing and self-driving cars can also optimize transportation needs and reduce traffic.

Multiple sectors, including building management, heating, ventilation, air conditioning systems, industry, information and communications technology, lighting, power grids, renewables, and transportation, can benefit from optimizing energy conservation through artificial intelligence. Individual actions such as using energy-efficient appliances, improving insulation and windows, adopting sustainable transportation, and utilizing renewable energy sources are emphasized in the plan for reducing energy consumption and carbon footprint. Other measures include conserving water, reducing food waste, planting trees for shade, and promoting energy conservation awareness. These changes in behavior can immediately save a substantial amount of oil, gas, and electricity annually. In general, energy-efficient building design, the electrification of transportation, and the increased use of renewable energy can reduce carbon emissions and improve energy sustainability. We must adopt intelligent building and mobility technologies, energy storage, and grid optimization systems to support this shift.
